# Supplementary material for: Access to public sector family planning services and modern contraceptive methods in South Africa: A qualitative evaluation from community and health care provider perspectives
Source: PLoS One. 2023 Mar 17;18(3):e0282996. doi: 10.1371/journal.pone.0282996 (PMC10022780; doi:10.1371/journal.pone.0282996)
Supplement: S1 Data — (PDF) [file pone.0282996.s001.pdf]

A HEALTH SECTOR AND COMMUNITY-BASED PARTICIPATORY APPROACH IN A HUMAN RIGHTS FRAMEWORK, TO INCREASE MET NEEDS FOR CONTRACEPTION: THE UPTAKE PROJECT

In-depth interview guide (Key stakeholders)

UMKHAKHA WEZEMPILO NENDLELA YOKUBAMBA IQHAZA KOMPHAKATHI NGENDLELA YEZINHLELO ZAMALUNGELO ABANTU, UKWANDISA IZIDINGO ESEZITHOLAKELE ZOKUVIKELA UKUKHULELWA: I UPTAKE PROJECT

Uhlelo lwengxoxo mbuzo ejulile (Abaqavile abamele umphakathi)

PID NUMBER:	LOCATION OF IDI:	DATE (DD/MMM/YY):	START TIME:	END TIME:	INTERVIEWER INITIALS:

[Read to participant/Fundela umbambiqhaza]
You have been invited here today to talk about the UPTAKE project.

<u>Purpose:</u>
I am interested in all your experiences, ideas, comments, suggestions and recommendations. This research is to help me understand how to best engage community members and know the experiences and challenges women and girls in the community face in accessing family planning and contraceptive services. This will help in sending feedback to policy makers and also be used to improve health services by identifying what you think are the challenges to accessing family planning and contraceptive services. All information will be treated with confidentiality.

<u>Explain the ground rules for the interview:</u>
We have just reviewed the consent form, which describes the study in detail and gives us permission to speak with you. You are not required to answer all of my questions, and you may skip any questions. As a reminder, I will use a digital recorder to record our conversation.

Do you have any questions before we begin the interview?

Umenyiwe lana namhlanje ukuzokhuluma nge-projethi ye-UPTAKE.

<u>Inhloso:</u>
Ngithanda ukwazi ngohlangabezane nakho, imibono, ukuphawula, iziphakamiso, Kanye nezincomo. Lolucwaningo luzongisiza ngiqonde ukuthi ngingawabandakanya kanjani ngokuzimisela amalunga omphakathi, ngiphinde ngazi abahlangabezane nakho nezinkinga abesifazane namantombazane ababhekane nazo emphakathini ekutholeni izinsiza zokuhlela umndeni nokuvikela ukukhulelwa. Lokhu kuzosiza ekuthumeleni okutholakele kubakhi benqubomgomo futhi kusetshenziswe ekwenzenincono izinsiza zezempilo ngokuhlonza lokhu ocabanga ukuthi izinselelo ekutholeni izinsiza zokuhlela umndeni kanye nokuvikela ukukhulelwa. Lonke ulwazi luzophathwa ngobumfihlo.

Chaza isishaya mthetho salengxoxo mbuzo:
Siqeda kubuyekeza iphepha lemvumo, elichaza ucwaningo kabanzi futhi lusinika imvume yokukhuluma nawe. Awubekelwe ukuthi uphendule yonke imibuzo yami, futhi ungeqa noma imuphi umbuzo. Njengesikhumbuzo, ngisosebenzisa isiqophamazwi ukuqopha ingxoxo yethu.

Ikhona imibuzo onayo ngaphambi kokuba siqale ingxoxo mbuzo?

[Turn on digital recorder.]

I am (INTERVIEWER NAME) interviewing (PARTICIPANT ID#) on [DATE] [START TIME]

**A. Background/*Imumva*** *[Please complete information in spaces below]/[Ngicela ugcwalise imininingwane ezikhaleni ngezansi]*		
1.	Sex:<br>*Ubulili:*	☐ Male/ *Owesilisa* ☐ Female/ *Owesifazane*
2.	Sector representing:<br>*Umkhakha awumele :*	
3.	Job title:<br>*(Please note this information could be an identifier, but individual information will be summarised in reports in order to protect your confidentiality.)*<br><br>Isihloko somsebenzi:<br>*(Ngicela uqaphele lolulwazi lungaba inkomba, kodwa imininingwane yomuntu ngamunye iyofinqwa kwimibiko ukuvikela ubumfihlo bakho.)*	
4.	Office/Department:<br><br>*Ihhovisi/umnyango:*	
5.	Highest educational level:<br><br>*Izinga lemfundo eliphezulu:*	
6	Number of years in current position:<br><br>*Mingaki iminyaka ukulesikhundla okuso:*	
7.	What are your primary responsibilities?<br><br>*Iliphi iqhaza olibambile?*	
8.	Age at last birthday<br><br>*Ubudala ngosukulozalwa eledlule*	________________ (age in years/*ubudala ngeminyaka*)

	**Main questions/*Imibuzo***	**Probes/*Buzisisa***
**Family planning knowledge, attitudes and practices**<br>***Ulwazi ngokuhlela umndeni, indlela abazizwa ngayo, nemikhuba***		
9.1	Please describe your understanding of family planning/ contraception services.<br><br>*Ngicela ungichazele ukuqonda kwakho ngezinsiza zokuhlela imindeni/ukuvikela ukukhulelwa.*	a. Describe the different family planning/contraceptive methods you know about.<br>*[Probe for different methods.]*<br><br>a. Chaza izindlela ezahlukene zokuhlela imindeni/ukuvikela ukukhulelwa owaziyo ngazo.<br>*[Buzisisa ngezindlela ezahlukene.]*
9.2	What is/are the most common method(s) of family planning/contraception <u>used</u> in your community?<br><br>*Iyiphi/yiziphi izindlela ezijwayelekile kakhulu zokuhlela umndeni/ukuvikela ukukhulelwa ezisetshenziswa emphakathini wakho?*	a. Why do you think this is the most common method(s)?<br>b. Do women and their partners change family planning/contraceptive methods?<br>*[Explore why, what methods and frequency of change.]*<br><br>a. Ucabanga ukuthi kungani kuyiyona esetshenziswa kakhulu?<br>b. Ingabe abesifazane nophathina babo bayazishintsha izindlela zokuhlela imindeni/ukuvikelwa ukukhulelwa?<br>Hlola kungani*, iziphi izindlela futhi izikhathi ezishintshana ngayo.*
9.3	What family planning/contraceptive methods are <u>available</u> (offered) in your community?<br><br>*Iziphi izindlela zokuhlela umndeni/ukuvimbela ukukhulelwa ezitholakalayo (ezinikelwayo) emphakathini wakho?*	a. What things make it difficult for people to get and use family planning/contraceptive methods?<br><br>*Probes: things about health services and health workers; other people's opinions about why certain people should/should not use family planning/contraception (especially teenagers and unmarried); whether or not people already have children; male partners' opinions; culture, religion; etc.*<br><br>a. Iziphi izinto ezenza kubenzima kubantu ukusebenzisa izindlela zokuhlula imindeni/ukuvikelwa ukukhulelwa?<br><br>*Buzisisa: izinto ngezinsiza zempilo nabasebenzi bezempilo; imibono yabanye abantu ngokuthi kungani abanye abantu kumele/kungamele basebenzise izindlela zokuhlela umndeni/ukuvikela ukukhulelwa (ikakhulukazi abantu abasebasha, Kanye nabangashadile) asebevele benazo noma abangenazo izingane; imibono yophathina besilisa, usiko, inkolo; njll.*

9.4	Who are the key people that support women and girls in choosing and using family planning and contraceptive methods?<br><br>*Obani abantu okuyibona bona abaseka abesifazane namantomabazane ekukhetheni nasekusebenziseni izindlela zokuhlela umnndeni Kanye nokuvikela ukukhulelwa?*	*Probe for*<br>• Partner<br>• Friends<br>• Parents<br>• Health workers<br>• Community leaders<br>• Religious leaders<br><br>*[Explore why these people are the most important.]*<br><br>a. Who makes the decision about using family planning/contraception in your community?<br><br>*Explore issues related to gender and power relations in family planning/contraceptive decision making.*<br><br>*Buzisisela*<br>• Uphathina<br>• Abangani<br>• Abazali<br>• Abasebenzi bezempilo<br>• Abaholi bomphakathi<br>• Abaholi bezenkolo<br><br>*Hlola kungani bebaluleke kakhulu labantu.*<br><br>a. Ubani othatha izinqumo ngokusebenzisa ukuhlelwa komndeni/nokuvikela ukukhulelwa emphakathini wakho?<br><br>*Hlola izindaba eziphathelene nobulili kanye namandla obudlelwano ekuthatheni izinqumo zokuhlela umndeni/nokuvikela ukukhulelwa.*

**Barriers and enablers to family planning/contraceptive access**<br>***Okuvimbelayo kanye nokuvumelayo (enablers) ekufinyeleleni kokuhlelwa komndeni/ukuvikela ukukhulelwa***		
10.1	Women, girls and families go through different experiences accessing family planning and contraceptive methods. What are the experiences of your community members in accessing these services?<br><br>*Abasifazane, amantombazane nemindeni bahlangabezana nezimo ezahlukene ekufinyeleleni ezindleleni zokuhlola imindeni Kanye nokuvikela ukukhulelwa.*<br>*Yikuphi amalunga omphakathi wakho ahlangabezana nakho ekufinyeleleni kulezizinsiza?*	a. What are some of the barriers and enablers to accessing family planning/contraceptive services in your area?<br><br>*Probe on:*<br>• access to healthcare facilities with family planning/contraceptive services,<br>• capacity of healthcare facilities,<br>• attitudes and knowledge of healthcare providers,<br>• availability of information about contraceptive/family planning services,<br>• culture, traditions, religion; etc.<br><br>a. Ikuphiphi okunye okuvimbelayo nokuvumelayo (enablers) ekufinyeleleni ezinsizeni zokuhlela umndeni/nokuvikela ukukhulelwa endaweni yakho?<br><br>*Buzisisa kulokhu:*<br>• Ukufinyelela ezikhungweni zomtholampilo ezinokuhlelwa komndeni/ukuvikela ukukhulelwa,<br>• Umthamo (capacity) wezikhungo zomtholampilo,<br>• Indlela ozizwa ngayo Kanye nolwazi lwabanikezeli bonakekelo lwezempilo,<br>• Ukutholakala kolwazi mayelana nezinsiza zokuvikela ukukhulelwa/ukuhlela umndeni,<br>• Usiko, amasiko (traditions), inkolo; njll.
10.2	What are the major sources of health information about family planning/contraceptive services in the community?<br><br>*Yimiphi imithombo emikhulu yolwazi lwezempilo ngezinsiza zokuhlela umndeni/ukuvikela ukukhulelwa emphakathini?*	Where does the community access their information about family planning and contraceptive services?<br><br>Umphakathi ulithola kuphi ulwazi mayelana nezinsiza zokuhlela umndeni Kanye nokuvikela ukukhulelwa?

10.3	Do the health care facilities in your area have the capacity to provide family planning/contraceptive services?<br><br>*Ingabe izikhungo zezempilo endaweni yakho zinawo amandla (capacity) okunikezela izinsiza zokuhlela umndeni/ukuvikela ukukhulelwa?*	*Probe on available resources, number of staff, operation hours, number of rooms available vs number of clients attending the facility, waiting time, etc.*<br><br>*a.* Do healthcare providers in your area have capacity to provide family planning/contraceptive services to all potential clients?<br><br>*Explore training received, number of years of practical experience, etc.*<br><br>*Buzisisa ngezinsiza zomsebenzi (resources) ezitholakalayo, inani labasebenzi, izikhathi zokusebenza, inani lezindlu ezikhona uqhathanisa nani leziguli (clients) elivakashela isikhungo, isikhathi sokulinda, njll.*<br><br>*a.* Ingabe abanikezi bonakekelo lwezempilo endaweni yakho banawo amandla (capacity) okunikezela izinsiza zokuhlela umndeni/nokuvikela ukukhulelwa kubobonke abangahle bebe iziguli?<br><br>*Hlola ukuqeqeshwa abakuthola, inani leminyaka yolwazi (practical experience) abanalo, njll.*
10.4	How would you describe the knowledge and attitudes of healthcare providers towards providing family planning and contraceptive services to their clients?<br><br>*Ungaluchaza kanjani ulwazi nendlela abazizwa ngayo abanikezeli bonakekelo lwezempilo ekunikezeni izinsiza zokuhlela umndeni kanye nokuvikela ukukhululwa ezigulini zabo?*	*Explore attitudes towards different categories of clients, including: Age of clients, marital status, sex of clients, occupation, rural vs urban, etc.*<br><br>*Hlola indlela abazizwa ngayo ezigululi ezahlukene, kubalwa: ubudala beziguli, isimo somshado, ubulili beziguli, umsebenzi, amakhaya eqhathaniswa nedolobha, njll.*
10.5	Where do you think family planning/contraceptive services should be made available to members of the community?<br><br>*Ucabanga ukuthi kumele zenziwe zitholakale kuphi izinsiza zokuhlela umndeni/ukuvikela ukukhulelwa kumalunga omphakathi?*	*Probe for facility type, service area, etc.*<br><br>a. Who do you think should be providing family planning/contraceptive services to the community?<br><br>*Buzisisa ngenhlobo yesikhungo, indawo yosizo, njll.*<br><br>a. Ucabanga ukuthi ubani okumele anikezele izinsiza zokuhlela umndeni/ukuvikela ukukhulelwa emphakathini?

10.6	Who are the major clients in your community who access family planning/contraceptive services?<br><br>*Obani iziguli okuyizonazona emphakathini wakho ezifinyelela ezinsizeni zokuhlela umndeni/ukuvikela ukukhulelwa?*	*Explore categories such as age, sex, marital status, rural vs urban, etc.*<br><br>*Hlola izigaba ezinjengobudala, ubulili, isimo somshado, amakhaya eqhathaniswa namadolobha, njll.*
10.7	What role do you think healthcare providers play in assisting young people to access contraceptive/family planning methods?<br><br>*Iliphi iqhaza ocabanga ukuthi abanikezeli bonakekelo lwezempilo bayalidlala ekusizeni abantu abasebancane bafinyelele ezindleleni zokuhlela umndeni/ukuvikela ukukhulelwa?*	a. What role do you think they should play?<br>b. Do you think that the family planning/contraceptive needs of the young people are met by the healthcare providers?<br>c. Do you think that healthcare providers and young people have the same goals/vision for providing and accessing contraceptive/family planning services?<br><br>a. Ucabanga ukuthi iyiphi indima okumele bayidlale?<br>b. Ucabanga ukuthi izidingo zabantu abasebancane zokuhlela umndeni/ukuvikela ukukhulelwa sezihlangabeziwe ngabanikezeli bonakekelo lwezempilo?<br>c. Ucabanga ukuthi abanikezeli bonakekelo lwezempilo nabantu abasebancane banempokophelo/imibono efanayo ekunikezeleni nasekufinyeleleni ezinsizeni zokuvikela ukukhulelwa/ukuhlela umndeni?
10.8	What advice would you give someone who is not using a contraceptive/family planning method or who may be interested in using or changing methods?<br><br>*Yisiphi iseluleko ongasinika umuntu ongazisebenzisi izindlela zezinsiza zokuvikela ukukhulelwa/ukuhlela umndeni noma mhlambe ongathanda ukusebenzisa noma ashintshe izindlela?*	*Probe for each:*<br>a. Someone who is not using a method<br>b. Someone who is interested in changing methods<br><br>*Buzisisela ngakunye:*<br>a. Umuntu ongasebenzisi ndlela<br>*b.* Umuntu ongathanda ukushintsha izindlela

10.9	What resources are available in your community to support women and girls in accessing family planning and contraceptive services?<br><br>*Iziphi izinsiza zokusebenza (resources) ezitholakalayo emphakathini wakho ukusiza abesifazane namantombazane ekufinyeleleni ezinsizeni zokuhlola umndeni Kanye nokuvikela ukukhulelwa?*	a. Are there any special services for women and girls wanting family planning and contraceptive services at your health facilities or in the local community?<br><br>*Explore what these services are, and where they are.*<br>*If there are services, explore their accessibility and whether they are used or not.*<br><br>a. Ngabe zikhona izinsiza ezikhethelekile zabesifazane namantombazane abafuna izinsiza zokuhlela umndeni Kanye nokuvikela ukukhulelwa esizindeni sezempilo noma emphakathini wendawo?<br><br>*Hlola ukuthi iziphi lezizinsiza, futhi zikuphi.*<br>*Uma zikhona izinsiza, hlola ukufinyelela (accessibility) kuzo nokuthi ziyasetshenziswa noma cha.*
10.10	How do you think decentralisation of services has affected (or could affect) community access to contraceptive/ family planning services?<br><br>*Ucabanga ukuthi ukusabalaliswa kwezinsiza kuwuthinte (kungawuthinta) kanjani umphakathi ekufinyeleleni ezinsizeni zokuvikela ukukhulelwa/ukuhlela umndeni?*	*Explore both positive and negative outcomes.*<br><br>*Hlola yomibili imiphumela emihle nemibi.*
**Quality of care**<br>***Izinga lonakekelo (Quality of care)***<br>***Note to interviewer: Remember to ask about quality (and not level) of care***		
11.1	How would you define good quality family planning/contraceptive services?<br><br>*Ungalichaza kanjani izinga lonakekelo oluhle lwezinsiza zokuhlela umndeni/nokuvikela ukukhulelwa?*	a. What constitutes good quality of care?<br><br>a. Yini eyakha izinga lonakekelo oluhle?

11.2	Some people say quality of care is influenced by issues such as available health care workers, integration of services, facility operation hours, number of rooms available, number of clients attending the facility, waiting time, etc. Which of these are important for you in describing good quality care?<br><br>*Abanye abantu bathi izinga lonakekelo oluhle luthelelwa izinto ezifana nokubakhona kwabasebenzi bonakekelo lwezempilo, ukuhlanganiswa kwezinsiza, izikhathi zokusebenza kwesizinda, inani lezindlu ezitholakalayo, inani leziguli ezifikayo esikhungweni, isikhathi sokulinda nokunye. Yikuphi kulokhu okubalulekile kuwena ekuchazeni izinga lonakekelo oluhle?*	
11.3	Are quality family planning/contraceptive services available to people in your community?<br><br>*Ingabe izinsiza ezisezingeni elihle zokuhlela umndeni/ukuvikela ukukhulelwa ziyatholakala kubantu emphakathini wakho?*	*Explore why or why not?*<br><br>*Hlola kungani, kungani kungenjalo?*
11.4	How do you think health care facilities could provide quality family planning/contraceptive services?<br><br>*Ucabanga ukuthi izizinda zonakekelo lwezempilo zingazinikezela kanjani izinsiza zokuhlela umndeni/ukuvikela ukukhulelwa okusezingeni eliphezulu?*	a. How do you think the services should be delivered?<br>b. Who should deliver the services?<br>c. What other information should be given to clients about family planning/contraceptive services?<br><br>d. Ucabanga ukuthi zingelethwa kanjani lezizinsiza?<br>e. Ubani okumele azilethe lezizinsiza?<br>f. Yiluphi olunye ulwazi okumele lunikezwe iziguli mayelana nezinsiza zokuhlela umndeni/nokuvikela ukukhulelwa?

<table>
<tr><td colspan="3">Community participation
Ukubamba iqhaza komphakathi</td></tr>
<tr><td>12.1</td><td>Community members and groups participate in different ways within the health system. How would you define community participation in this community?

Amalunga omphakathi namaqembu abambiqhaza ngezindlela ezahlukene phakathi ohlelweni lwezempilo. Ungakuchaza kanjani ukubamba iqhaza komphakathi kulomphakathi?</td><td></td></tr>
<tr><td>12.2</td><td>What are some of the existing community participation activities in this area?

Imiphi eminye imisebenzi ekhona umphakathi obambe kuyo iqhaza kulendawo?</td><td>a. Who participates in these activities? And how?
b. How does the community feel about these activities?
c. What community participation activities work and which ones don’t work? Explore why – probe for issues of age, religion and cultural acceptability of community participation.
d. What are some of the challenges to community participation in your area? (Also explore if no community participation activities in the area).
e. Who should participate if a project is created on family planning and contraceptive service in this community? How should they participate?

a. Ubani obamba iqhaza kulemisebenzi? Futhi kanjani?
b. Umphakathi uzizwa kanjani ngalemisebenzi?
c. Imiphi imsebenzi (activities) esebenzayo nengasebenzi umphakathi obamba kuyo iqhaza? [Hlola kungani- buzisisa ezindabeni ezifana nobudala, inkolo kanye nokwamukeleka ngokosiko lokubamba iqhaza komphakathi.]
d. Iziphi ezinye zezinselelo zokubamba iqhaza komphakathi endaweni yakho? (Phinda uhlole ukuthi ayikho yini imisenzi (activities) umphakathi obamba kuyo iqhaza endaweni).
e. Ubani okumele abambe iqhaza emphakathini uma iprojethi yakhiwe ezinsizeni zokuhlela umndeni kanye nokuvikela ukukhulelwa kulomphakathi? Futhi kumele balibambe kanjani iqhaza?</td></tr>
</table>

12.3	What role do you play in facilitating community participation in your area?<br><br>*Iyiphi indima oyidlalayo ekuqhubeni (facilitating) ukubamba iqhaza komphakathi endaweni yakho?*	How does your role in the community relate to health and family planning/contraceptive services access for the community?<br><br>Ingabe iqhaza lakho emphakathini lihlangana kanjani nokufinyelela kwezezempilo kanye nezinsiza zokuhlela umndeni/ukuvikela ukukhulelwa?
12.4	How do you think community participation can be used to improve access to family planning/contraceptive services?<br><br>*Ucabanga ukuthi ukubamba iqhaza komphakathi kungasetshenziswa kanjani ekwenzeni ncono ukufinyelela ezinsizeni zokuhlela umndeni/ukuvikela ukukhulelwa?*	a. What are your recommendations for improving community engagement with healthcare providers when accessing family planning/contraceptive services? *Probe for consideration of age (teenagers vs older women, married vs unmarried, rural vs urban, etc.)*<br><br>a. Yikuphi ongakuncoma ekuthuthukiseni ukuzibandakanya komphakathi nabanikezeli bokunakekela kwezempilo ekufinyeleleni ezinsizeni zokuhlela umndeni/nokuvikela ukukhulelwa? *Buzisisa ngokucabangela ubudala (intsha iqhathaniswa nabesifazane abadala, abashadile beqhathaniswa nabangashadile, amakhaya eqhathaniswa nedolobha, nokunye)*
12.5	What role do you think the community should play to improve future access to family planning/contraceptive services?<br><br>*Ucabanga ukuthi iyiphi indima umphakathi okumele uyidlale ukwenza kancono ukufinyelela okuzayo ezinsizeni zokuhlela umndeni/ukuvikela ukukhulelwa?*	a. How can the community be engaged in future interventions for improved uptake of family planning/contraceptive services?<br>b. What could these interventions be? *Explore.*<br><br>a. Umphakathi ungabandakanyeka kanjani ekungeneleleni okuzayo ukwenza kangcono izinsiza ze-uptake yokuhlela umndeni/ukuvikela ukukhulelwa?<br>b. Kungaba yikuphi lokhukungenelela? *Hlola.*

**Conclusion**<br>***Isiphetho***		
13.1	Do you have anything else that you would like to tell us about family planning/contraception and community participation before we end?<br><br>*Kukhona okunye onakho ongathanda ukusitshela khona mayelana nokuhlela umndeni/ukuvikela ukukhulelwa Kanye nokubamba iqhaza komphakathi ngaphambi kokuba siqede?*	

This is the end of our discussion. Thank you for your time.
Sekuyisiphetho sengxoxo yethu lesi. Ngiyabonga ngesikhathi sakho.
