# Supplementary material for: Access to public sector family planning services and modern contraceptive methods in South Africa: A qualitative evaluation from community and health care provider perspectives
Source: PLoS One. 2023 Mar 17;18(3):e0282996. doi: 10.1371/journal.pone.0282996 (PMC10022780; doi:10.1371/journal.pone.0282996)
Supplement: S3 Data — (PDF) [file pone.0282996.s003.pdf]

A HEALTH SECTOR AND COMMUNITY-BASED PARTICIPATORY APPROACH IN A HUMAN RIGHTS FRAMEWORK, TO INCREASE MET NEEDS FOR CONTRACEPTION: THE UPTAKE PROJECT

Focus Group Discussion guide (Males)

UMKHAKHA WEZEMPILO NENDLELA YOKUBAMBA IQHAZA KOMPHAKATHI NGENDLELA YEZINHLELO ZAMALUNGELO ABANTU, UKWANDISA IZIDINGO ESEZITHOLAKELE ZOKUVIKELA UKUKHULELWA: I UPTAKE PROJECT

Uhla lwengxoxo yeqembu ehleliwe (Abesilisa)

FOCUS GROUP ID NUMBER:	LOCATION OF FGD:	DATE (DD/MMM/YY):	START TIME:	END TIME:	MODERATOR INITIALS:

[Read to participant/ Fundela umbambiqhaza]
You have been invited here today to talk about the UPTAKE Project.

Purpose
We are interested in all your experiences, ideas, comments, suggestions and recommendations. This research is to help us understand how to best engage community members like yourself and know the experiences and challenges the community faced and are still facing in accessing family planning and contraceptive services. This will help in sending feedback to policy makers and also be used to improve health services by identifying what you think are the challenges to accessing family planning and contraceptive services. All information will be treated with confidentiality.

Explain the ground rules for discussion
This is a friendly discussion so; there is no right or wrong answer. Everyone should relax and feel free to discuss his opinion. We would like to have one person talk at a time and when one person is talking, there will be no interruption until the person has ended then the next person will be allowed to air his view. There will be no side discussions. Anyone can contribute to the discussion at any time. Every one of you should feel free to disagree or agree in a cordial manner. Please remember that what we discuss here today is confidential, do not discuss private information disclosed in this group with others outside of this group. Please turn cell phones on to silent so as not to disrupt the discussion. We will spend about one and a half to two hours for the discussion and some refreshment will be served at the end of the discussion.

We have just reviewed the consent form, which describes the study in detail and gives us permission to speak with you. As a reminder, you are not required to answer all of my questions, and you may skip any questions. As a reminder, we will use a digital recorder to record our conversation.

Do you have any questions before we begin the discussion?

Umenyiwe lana namhlanje ukuzokhuluma nge-projethi ye-UPTAKE.

<u>Inhloso</u>
Sithanda ukwazi ngohlangabezane nakho, imibono, ukuphawula, imibono eyisisombululo, kanye nezincomo. Lolucwaningo luzosisiza ngiqonde ukuthi angawabandakanyeka kanjani ngokuzimisela amalunga omphakathi, siphinde sazi ngezinkinga abesifazane namantombazane ahlangene nazo nasahlangana nazo emphakathini ekutholeni izinsiza zokuhlela umndeni Kanye nokuvikela ukukhulelwa. Lokhu kuzosiza ekuthumeleni okutholakele kubakhi benqubomgomo futhi kusetshenziswe ekwenzenincono izinsiza zezempilo ngokuhlonza lokhu ocabanga ukuthi izinselelo ekutholeni izinsiza zokuhlela umndeni Kanye nokuvikela ukukhulelwa. Lonke ulwazi luzophathwa ngobumfihlo.

<u>Chaza isisuka mthetho salengxoxo</u>
Lena yingxoxo enobungani ngakhoke; ayikho impendulo okuyiyona noma okungeyona. Wonke umuntu akanethezeke futhi akhululeke ukuxoxa umbono wakhe. Sizocela ukuba nomuntu oyedwa okhulumayo ngesikhathi, ngeke kube nokuphazamiseka aze aqede umuntu bese omunye umuntu olandelayo uzobe esevumelekile ukuphakamisa umbono wakhe. Ngeke kube khona izingxoxo eceleni. Noma ubani angabeka umbono engxoxweni noma ngasiphi isikhathi. Nonke ngingakhululeka ukuphikisana noma ukuvumelana ngendlela enhle. Sicela ukhumbule ukuthi esikuxoxa la namhlanje kuyimfihlo, ungaluxoxi ulwazi olukhishwe kuleliqembu nabanye abangaphandle kuleliqembu. Sicela ucime umakhala ekhukhwini khona ungeke uphazamise ingxoxo. Sizothatha mhlambe ihora nohhafu kuya emahoreni amabili futhi neziphuzo ziyotholakala ekupheleni kwengxoxo.

Siqeda kubuyekeza iphepha lemvumo, elichaza ucwaningo kabanzi futhi lusinika imvume yokukhuluma nawe. Njengesikhumbuzo, awubekelwe ukuthi uphendule yonke imibuzo yami, futhi ungeqa noma imuphi umbuzo. Njengesikhumbuzo, sizosebenzisa isiqophamazwi ukuqopha ingxoxo yethu.

Ikhona imibuzo onayo ngaphambi kokuba siqale ingxoxo?

[Turn on digital recorder.]

I am (MODERATOR NAME) interviewing (FOCUS GROUP ID#) on [DATE] [START TIME]

	Main question/*Imibuzo*	Probe/*Buzisisa*
**Family planning knowledge, attitudes and practices**<br>***Ulwazi ngokuhlela umndeni, indlela abazizwa ngayo, nemikhuba***		
1.1	Please describe your understanding of family planning (or contraception) services.<br><br>*Ngicela ungichazele ukuqonda kwakho ngezinsiza zokuhlela umndeni (noma ukuvikela ukukhulelwa)*	a. Describe the different family planning/contraceptive methods you know about. *Probe for different methods.*<br>b. In your opinion, how well or poorly do family planning/contraceptive methods work to prevent pregnancy?<br>c. Who do you think should use family planning/contraceptive services? *Probe for marital status, parity, age (including teenagers), etc.*<br>d. What do men <u>like</u> about family planning/contraceptives? (Apart from preventing pregnancy, do they have other positive effects?) *Probe for specific advantages of different methods.*<br>e. What do men <u>not like</u> about family planning/contraceptives? Why? *Probe for specific things they don't like about different methods.*<br><br>*Explore issues related to gender and perceived benefits/disadvantages.*<br><br>f. When choosing their family planning/contraceptive method, do people think about whether it prevents STIs/HIV?<br><br>a. Chaza ngezinhlobo ezahlukene zokuhlela umndeni/ukuvikela ukukhulelwa owaziyo ngazo? *Buzisisela izindlela ezahlukene.*<br>b. Ngokwakho ukubona, izindlela zokuhlela umndeni/ukuvikela ukukhulelwa zikuvikela kahle noma kabi kanjani ukukhulelwa?<br>c. Ucabanga ukuthi ubani okumele asebenzise izinsiza zokuhlela umndeni/ukuvikela ukukhulelwa? *Buzisisela isimo somshado, isilinganiso, ubudala (kubalwa nentsha), njll.*<br>*d.* Yini abesilisa <u>abakuthandayo</u> ngokusebenzisa ukuhlela umndeni/ukuvikela ukukhulelwa? (Ngaphandle kokuvimbela ukukhulelwa, banayo eminye imiphumela emihle?) *Buzisisa ngobuhle obuthize bezindlela ezahlukene?*<br>*e.* Yini abesilisa <u>abangakuthandi</u> ngokusebenzisa ukuhlela umndeni/ukuvikela ukukhulelwa? Kungani? *Buzisisa ngezinto ezithize abangazithandi ngezindlela ezahlukene?*<br><br>*[Hlola izindaba ezihlobene nobulili Kanye zezinzuzo esezibonakele/ububi bayo.]*<br><br>f. Uma abantu bekhetha indlela yokuhlela umndeni/ukuvikela ukukhulelwa, ingabe bayacabanga ukuthi iyasivikela isifo esithathelana ngocansi/isandulela ngculaza.

1.2	What is/are the most common method(s)of family planning/contraception used in your community?<br><br>*Yini/yiziphi izindlela ezijwayelekile zokuhlela umndeni/ukuvikela ukukhulelwa ezisetshenziswa emphakathini wakho?*	a. Why do you think this is the most common method(s)?<br>b. Which is <u>your and your partner's</u> preferred method and why?<br><br>*If participants and their partners don't use family planning/ contraception, explore why.*<br><br>*Facilitator to make a note of numbers who don't use family planning/contraception methods.*<br><br>a. Ucabanga ukuthi kungani kuyizindlela ezisetshenziswa kakhulu?<br>b. Iyiphi <u>eyakho indlela kanye nekaphathina</u> eniyincamelayo futhi kungani?<br><br>*Uma ababambiqhaza kanye nophathina babo bengakusebenzisi ukuhlela umndeni/ukuvikela ukukhulelwa, hlola kungani.*<br><br>*Umphathi ngxoxo enze amanothi ezinombolo zalabo abangazisebenzisi izindlela zokuhlela umndeni/ukuvikela ukukhulelwa.*
1.3	What family planning/contraceptive methods are available in your community?<br><br>*Iziphi izindlela zokuhlela umndeni/ukuvimbela ukukhulelwa ezitholakalayo emphakathini wakho?*	a. What things make it difficult for young people to get and use family planning/contraceptive methods to prevent pregnancy when they want to use them?<br><br>*Probes: things about health services and health workers; other people's opinions about young women using family planning/contraception (especially teenagers and unmarried women); whether or not people already have children; male partners' opinions, etc.*<br><br>b. Are family planning/contraceptive services freely available at your clinic?<br><br>a. Iziphi izinto ezenza kubenzima kubantu abancane ukuthola nokusebenzisa izinsiza zokuhlela umndeni/ukuvikela ukukhulelwa ukuvimbela ukukhulelwa mabefuna ukuzisebenzisa?<br><br>*Buzisisa: izinto ngezinsiza zempilo nabasebenzi bezempilo; imibono yabantu ngabantu besifazane abasebancane abasebenzisa izindlela zokuhlela umndeni/ukuvikela ukukhulelwa (ikakhulukazi abantu abasebasha, Kanye nabesifazane abangashadile) asebevele benazo noma abanazo izingane; imibono yophathina besilisa, njll.*<br><br>b. Ingabe izinsiza zokuhlela umndeni/ukuvikela ukukhulelwa zitholakala mahhala emtholampilo wakho?

1.4	Is there a family planning/contraceptive method you and your partner wish to try but it is not available at your clinic?<br><br>*Ingabe ikhona indlela yokuhlela umndeni/ukuvikela ukukhulelwa enifisa ukuyizama wena nophathina wakho kodwa ayitholakali emtholampilo wakho?*	*Yes/No?*<br>*Moderator to ask each participant, and record responses.*<br><br>*Explore what these methods are.*<br><br>*Yebo/Cha?*<br>*Umphathi ngxoxo abuze umbambiqhaza ngamunye, aphinde aqophe izimpendulo.*<br><br>*Hlola iziphi lezindlela lezi.*
1.5	Who are the most important people in supporting women and girls in choosing and using family planning and contraceptive methods?<br><br>*Obani abantu ababaluleke khakhulu ekusizeni abasifazane Kanye namantombazane ekukhetheni Kanye nasekusebenziseni izindlela zokuhlela umndeni kanye nokuvikela ukukhulelwa?*	*Probe for*<br>• Partner<br>• Friends<br>• Parents<br>• Health workers<br>• Community leaders<br>• Religious leaders<br><br>*Explore why these people are the most important.*<br><br>a. Do you discuss family planning/contraception with your partner(s)?<br>b. Who makes the decision about family planning/contraception in your relationships?<br><br>*Buzisisela*<br>• Uphathina<br>• Abangani<br>• Abazali<br>• Abasebenzi bezempilo<br>• Abaholi bomphakathi<br>• Abaholi bezenkolo<br><br>*Hlola kungani bebaluleke kakhulu labantu.*<br><br>a. Niyaxoxisana ngokuhlela umndeni/ukuvikela ukukhulelwa nophathina wakho/bakho?<br>b. Ubani othatha izinqumo ngokuhlela umndeni/ukuvikela ukukhulelwa ebudlelwaneni benu?

**Barriers and enablers to family planning access**<br>***Okuvimbelayo Kanye nokuvumelayo (enablers) ekufinyeleleni ekuhleleni umndeni***		
2.1	Women, girls and families go through different experiences using family planning and contraceptive methods. What are some of the experiences you or your friends have encountered?<br><br>*Abesifazane, amantombazane Kanye nemindeni bahlangabezana nezimo ezahlukene ekusebenziseni izindlela zokuhlela umndeni Kanye nokuvikela ukukhulelwa. Iziphi ezinye zezimo ohlanganbezane nazo noma abanganibakho abahlangane nazo?*	Do women and their partners change family planning/contraceptive methods used? *Explore why, and what methods.*<br><br>Ingabe abesifazane nophathina babobayashintsha indlela yokuhlela umndeni/ukuvikela ukukhulelwa abakusebenzisayo? *Hlola kungani, futhi iziphi izindlela.*
2.2	Can we discuss your experiences accessing family planning and contraceptive services?<br><br>*[Note to interviewer: participants may or may not have experiences accessing these services, and some may be limited to condom access, but allow participants to express their views without leading them].*<br><br>*Singaxoxisana ngohlangabezane nakho ekutholeni izinsiza zokuhlela umndeni kanye nokuvikela ukukhulelwa?*	a. How did you know about the method/s available?<br>b. Did you seek out these family planning/contraceptive services, or were you approached to use these services?<br>c. How was family planning/contraception approached and discussed with you? *[if participant refers to healthcare provider, explore questions in 2.3 below]*<br>d. Who are your main sources of health information about family planning/contraceptive use, risks?<br><br>*Give each participant a paper and pen, ask participants:* Please draw a picture of where you/your partner access your family planning/contraceptive services from, in relation to your home. Include estimates of distance/time taken to get to the facilities.<br><br>a. Waze kanjani ngendlela/izindlela ezitholakalayo?<br>b. Ingabe wazifuna lezinsiza zokuhlela umndeni/ukuvikela ukukhulelwa, noma khona oweza kuwe ngalezinsiza?<br>c. Ingabe waqondwa kanjani futhi kwaxoxwa kanjani ngokuhlela umndeni/ukuvikela ukukhulelwa nawe? *[uma ababambiqhaza bebhekisa kumnikezeli wonakekelo lwezempilo, hlola imibuzo ku 2.3 ngezansi]*<br>d. Obani imithombo yolwazi lwezempilo ngobungozi bokusebenzisa ukuhlela umndeni/ukuvikela ukukhulelwa?<br><br>*Nikeza umbambiqhaza ngamunye iphepha nepeni, cela ababambiqhaza:*<br>Sicela udwebe isithombe salapho wena nophathina wakho nithola khona izinsiza zokuhlela umndeni/ukuvikela ukukhulelwa, ekuhlobeni nekhaya lakho. Bala nokulinganisa ubude (distance)/isikhathi esithathwayo ukufinyelela esikhungweni.

2.3	Can we discuss your experiences with health care providers when seeking or being spoken to about family planning/contraceptive services?<br><br>*[Note to interviewer: Participants may not have experiences with healthcare providers, if not, do not focus on probes].*<br><br>*Singaxoxa ngowahlangabezana nakho nabanikezeli bonakekelo lwezempilo ngesikhathi ufuna noma kukhulunywa nawe ngezinsiza zokuhlela umndeni/ukuvikelwa ukukhulelwa?*	*Explore attitude of healthcare providers during visit.*<br><br>a. Did you initiate the discussion of family planning/contraceptive services? How did the health care provider react?<br>b. If the healthcare provider initiated the discussion, how did they go about it?<br>c. Did you ask questions and how did the health worker respond to your queries?<br>d. Were your concerns effectively addressed by the health care provider?<br><br>*Hlola indlela abazizwa ngayo abanikezeli bonakekelo lwezempilo ngesikhathi uvakashile.*<br><br>a. Ingabe wayiqala ingxoxo yezinsiza zokuhlela umndeni/ukuvikela ukukhulelwa? Ingabe umnikezeli wonakekelo lwezempilo wenzenjani? (react)<br>b. Uma umnikezeli wonakekelo lwezempilo ayiqala ingxoxo, wayiqala kanjani?<br>c. Ingabe wayibuza imibuzo, futhi umnikezeli wonakekelo lwezempilo waphendula kanjani emibuzweni yakho?<br>d. Ingabe ukukhathazeka kakho kwadlulwa kukho ngendlela ngumnikezeli wonakekelo lwezempilo? (effectively)
2.4	Are there any religious or cultural barriers or facilitators to accessing family planning/contraceptive services?<br><br>*Ingabe kukhona ukuvimbela kwenkolo noma kosiko noma okwenza kubelula ukufinyelela ezinsizeni zokuhlela umndeni/ukuvikela ukukhulelwa?*	*Explore barriers and facilitators at both religious and cultural level.*<br><br>*a. If barriers were reported:*<br>How can these be overcome?<br><br>*Hlola okuvimbelayo nokwenza kubelula kukho kokubili ezingeni lenkolo Kanye nokosiko.*<br><br>*a. Uma ukuvimbela kwakubikiwe:*<br>Kunganqotshwa kanjani lokhu?

2.5	What role do you think healthcare providers play in assisting young people to access contraceptive/family planning methods?<br><br>*Iliphi iqhaza ocabanga ukuthi abanikezeli bonakekelo lwezempilo bayalidlala ekusizeni abantu abasebancane bafinyelele ezinsizeni zezindlela zokuhlela umndeni/ukuvikela ukukhulelwa?*	a. What role do you think they should play?<br>b. Do you think that the family planning/contraceptive needs of the young people are met by the healthcare providers?<br>c. Do you think that healthcare providers and young people have the same goals/vision for providing and accessing contraceptive/family planning services?<br><br>a. Ucabanga ukuthi iyiphi indima okumele bayidlale?<br>b. Ucabanga ukuthi izidingo zabantu abasebancane zokuhlela umndeni/ukuvikela ukukhulelwa sezihlangabeziwe ngabanikezeli bonakekelo lwezempilo?<br>c. Ucabanga ukuthi abanikezeli bonakekelo lwezempilo nabantu abasebancane banezifezo/umbono efanayo ekunikezeleni nasekufinyeleleni ezinsizeni zokuvikela ukukhulelwa/ukuhlela umndeni?
2.6	What advice would you give someone else who is not using a contraceptive/family planning method or who may be interested in using or changing methods?<br><br>*Yisiphi iseluleko ongasinika umuntu ongazisebenzisi izindlela zezinsiza zokuvikela ukukhulelwa/ukuhlela umndeni noma mhlambe ongathanda ukusebenzisa noma ashintshe izindlela?*	*Probe for each:*<br>*a. Someone who is not using a method*<br>*b. Someone who is interested in changing methods*<br><br>*Buzisisela ngakunye:*<br><br>*a. Umuntu ongasebenzisi ndlela*<br>*b. Umuntu ongathanda ukushintsha izindlela*
2.7	What resources are available in your community to support women and girls in accessing family planning and contraceptive services?<br><br>*Iziphi izinsiza zokusebenza (resources) ezitholakalayo emphakathini wakho ukusiza abesifazane namantombazane ekufinyeleleni ezinsizeni zokuhlola umndeni Kanye nokuvikela ukukhulelwa?*	
**Quality of care**<br>***Izinga lonakekelo (Quality of care)***<br>***Note to facilitator: Remember to ask about quality (and not level) of care***		
3.1	How would you define good quality family planning/contraceptive services?<br><br>*Ungalichaza kanjani unakekelo oluhle lwezinsiza zokuhlela umndeni/nokuvikela ukukhulelwa?*	What constitutes good quality of care?<br><br>Yini eyakha unakekelo olusezingeni oluhle?

3.2	How would you like to receive quality family planning/contraceptive services?<br><br>*Ungathanda ukuzamukela kanjani izinsiza ezisezingeni zokuhlela umndeni/ukuvikela ukukhulelwa?*	a. How would you like to access the services?<br>b. Where would you like to access the services from?<br>c. Who would you like to access the services from?<br>d. What other information do you need about family planning services?<br><br>a. Ungathanda ukufinyelela/ukuzithola kanjani kulezinsiza?<br>b. Ungathanda ukuzithola kuphi khona lezinsiza?<br>c. Ubani ongathanda ukuzithola kuye lezinsiza?<br>d. Yiluphi olunye ulwazi oludingayo ngezinsiza zokuhlela umndeni?
3.3	Are quality family planning/contraceptive services available to people in your community?<br><br>*Ingabe izinsiza ezisezingeni zokuhlela umndeni/ukuvikela ukukhulelwa ziyatholakala kubantu emphakathini wakho?*	*Explore why or why not?*<br><br>*Hlola kungani, kungani kungenjalo?*
**Community participation**<br>***Ukubamba iqhaza komphakathi***		
4.1	Community members and groups participate in different ways within the health system. How would you define community participation in this community?<br><br>*Amalunga omphakathi namaqembu abamba iqhaza ngezindlela ezahlukene phakathi ohlelweni lwezempilo. Ungakuchaza kanjani ukubamba iqhaza komphakathi kulomphakathi?*	*Explore group understanding vs individual opinions?*<br><br>*Hlola ukuqonda kweqembu uqhathanisa namuntu ngamunye?*

4.2	What are some of the existing community participation activities in this area?<br><br>*Imiphi eminye imisebenzi ekhona umphakathi ebambe kuyo iqhaza kulendawo?*	a. Who participates in these activities? And how?<br>b. How does the community feel about these activities?<br>c. What community participation activities work and which ones don't work? *Explore why– probe for issues of age, religion and cultural acceptability of community participation.*<br>d. What are some of the challenges to community participation in your area? *Also explore if no community participation activities in the area.*<br>e. Who should participate if a project is created on family planning and contraceptive service in this community? How should they participate?<br><br>a. Ubani obamba iqhaza kulemisebenzi? Futhi kanjani?<br>b. Umphakathi uzizwa kanjani ngalemisebenzi?<br>c. Imiphi imsebenzi esebenzayo nengasebenzi umphakathi obamba kuyo iqhaza? *Hlola kungani-buzisisa ezindabeni ezifana nobudala, inkolo kanye nokwamukeleka ngokosiko kokubamba iqhaza komphakathi.*<br>d. Iziphi ezinye zezinselelo zokubamba iqhaza komphakathi endaweni yakho? *(Phinda uhlole ukuthi ayikho yini imisenzi umphakathi obamba kuyo iqhaza endaweni).*<br>e. Ubani okumele abambe iqhaza emphakathini uma iprojethi yakhiwe ezinsizeni zokuhlela umndeni Kanye nokuvikela ukukhulelwa kulomphakathi? Kumele balibambe kanjani iqhaza?
4.3	How do you think community participation can be used to improve access to family planning/contraceptive services?<br><br>*Ucabanga ukuthi ukubamba iqhaza komphakathi kungasetshenziswa kanjani ekwenzeni ncono ukufinyelela ezinsizeni zokuhlela umndeni/ukuvikela ukukhulelwa?*	What are your recommendations for improving community engagement with healthcare providers when accessing family planning/contraceptive services?<br><br>*Probe for consideration of age (teenagers vs older women, married vs unmarried, rural vs urban, etc.)*<br><br>Yikuphi ongakuncoma ekuthuthukiseni ukuzibandakanya komphakathi nabanikezeli bonakekelo lwezempilo ekufinyeleleni ezinsizeni zokuhlela umndeni/nokuvikela ukukhulelwa?<br><br>*Buzisisa ngokucabangela ubudala (intsha iqhathaniswa nabesifazane abadala, abashadile beqhathaniswa nabangashadile, amakhaya eqhathaniswa nedolobha, nokunye)*

4.4	What role do you think the community should play to improve future access to family planning/contraceptive services?<br><br>*Ucabanga ukuthi iyiphi indima umphakathi okumele uyidlale ukuthuthukisa ukufinyelela okuzayo ezinsizeni zokuhlela umndeni/ukuvikela ukukhulelwa?*	a. How can the community be engaged in future interventions for improved uptake of family planning/contraceptive services?<br>b. What could these interventions be?<br>*Explore.*<br><br>a. Umphakathi ungabandakanyeka kanjani ekungeneleleni okuzayo bokuthuthukisa izinsiza ze-uptake yokuhlela umndeni/ukuvikela ukukhulelwa?<br>b. Kungabe yikuphi lokhukungenelela?<br>*Hlola.*
**Conclusion**<br>***Isiphetho***		
5.1	Do you have anything else that you would like to tell us about family planning/contraception and community participation before we end?<br><br>*Kukhona okunye onakho ongathanda ukusitshela khona mayelana nokuhlela umndeni/ukuvikela ukukhulelwa Kanye nokubamba iqhaza komphakathi ngaphambi kokuba siqede?*	

This is the end of our discussion. Thank you for your time.
Sekuyisiphetho sengxoxo yethu lesi. Ngiyabonga ngesikhathi sakho.
