# Supplementary material for: Access to public sector family planning services and modern contraceptive methods in South Africa: A qualitative evaluation from community and health care provider perspectives
Source: PLoS One. 2023 Mar 17;18(3):e0282996. doi: 10.1371/journal.pone.0282996 (PMC10022780; doi:10.1371/journal.pone.0282996)
Supplement: S4 Data — (PDF) [file pone.0282996.s004.pdf]

A HEALTH SECTOR AND COMMUNITY-BASED PARTICIPATORY APPROACH IN A HUMAN RIGHTS FRAMEWORK, TO INCREASE MET NEEDS FOR CONTRACEPTION: THE UPTAKE PROJECT

Focus Group Discussion guide (Health Care Providers)

UMKHAKHA WEZEMPILO NENDLELA YOKUBAMBA IQHAZA KOMPHAKATHI NGENDLELA YEZINHLELO ZAMALUNGELO ABANTU, UKWANDISA IZIDINGO ESEZITHOLAKELE ZOKUVIKELA UKUKHULELWA: I UPTAKE PROJECT

Uhlelo lwengxoxo yeqembu oluhleliwe (Abanikezeli Bonakekelo Lwezempilo)

FOCUS GROUP ID NUMBER:	LOCATION OF FGD:	DATE (DD/MMM/YY):	START TIME:	END TIME:	MODERATOR INITIALS:

<u>Introduction</u>
We welcome all of you to this discussion. We are happy that you have spared some time to come and have this discussion. Let us start first by introducing ourselves. My name is …..and I will be leading the discussion. With me is……………………. who will be writing down some notes.

[Read to participant]
You have been invited here today to talk about the UPTAKE Project.

<u>Isingeniso</u>
Siyanamukela nonke kulengxoxo. Siyajabula ukuthi nigcine(spared) isikhathi ukuthi nize nizoba nalengxoxo. Asiqale ngokuqala sizethule. Igama lami ngu ……………………futhi ngizobe ngiqhuba ingxoxo. Ohamba nami u……………………..ozobe ebhala phansi amanothi.

[Fundela umbambiqhaza]
Umenyiwe lana namhlanje ukuzokhuluma nge-projethi ye-UPTAKE.
<u>Purpose</u>
We are interested in all your experiences, ideas, comments, suggestions and recommendations. This research is to help us understand how to best engage health care providers like yourself and know the experiences and challenges you as health care providers face in providing family planning and contraceptive services and information to your clients. This will help in sending feedback to policy makers and also be used to improve health services by identifying what you think are the challenges to provision of family planning and contraceptive services and information. All information will be treated with confidentiality.

<u>Inhloso:</u>
Sithanda ukwazi ngohlangabezane nakho, imibono, ukuphawula, iziphakamiso, kanye nezincomo. Lolucwaningo luzosisiza siqonde ukuthi singababandakanya kanjani abasebenzi bonakekelo lwezempilo ngokuzimisela, njengani nje, siphinde sazi enihlangabezane nakho kanye nezinkinga nina njengabanikezeli

bonakekelo lwezempilo ekunikezeleni izinsiza zokuhlela umndeni nokuvikela ukukhulelwa kanye nolwazi kwiziguli. Lokhu kuzosiza ekuthumeleni okutholakele kubakhi benqubomgomo futhi kusetshenziswe ekwenzenincono izinsiza zezempilo ngokuhlonza lokhu ocabanga ukuthi izinselelo ekutholeni izinsiza zokuhlela umndeni Kanye nokuvikela ukukhulelwa. Lonke ulwazi luzophathwa ngobumfihlo.

Explain the ground rules for discussion

This is a friendly discussion so; there is no right or wrong answer. Everyone should relax and feel free to discuss his/her opinion. We would like to have one person talk at a time and when one person is talking, there will be no interruption until the person has ended then the next person will be allowed to air his/her view. There will be no side discussions. Anyone can contribute to the discussion at any time. Every one of you should feel free to disagree or agree in a cordial manner. Please remember that what we discuss here today is confidential, do not discuss private information disclosed in this group with others outside of this group. Please turn cell phones on to silent so as not to disrupt the discussion. We will spend about one and a half to two hours for the discussion and some refreshment will be served at the end of the discussion.

We have just reviewed the consent form, which describes the study in detail and gives us permission to speak with you. You are not required to answer all of my questions, and you may skip any questions. As a reminder, we will use a digital recorder to record our conversation.

Do you have any questions before we begin the discussion?

Chaza isisuka mthetho salengxoxo

Lena yingxoxo enobungani ngakhoke; ayikho impendulo okuyiyona noma okungeyona. Wonke umuntu akanethezeke futhi akhululeka ukuxoxa umbono wakhe. Sizocela ukuba nomuntu oyedwa okhulumayo ngesikhathi, ngeke kube nokuphazamiseka aze aqede umuntu bese omunye umuntu olandelayo uzobe esevumelekile ukuphakamisa umbono wakhe. Ngeke kube khona izingxoxo eceleni. Noma ubani angabeka umbono engxoxweni noma ngasiphi isikhathi. Nonke ngingakhululeka ukuphikisana noma ukuvumelana ngendlela enhle. Sicela ukhumbule ukuthi esikuxoxa la namhlanje kuyimfihlo, ungaluxoxi ulwazi olukhishwe kuleliqembu nabanye abangaphandle kuleliqembu. Sicela ucime umakhala ekhukhwini khona ungeke uphazamise ingxoxo. Sizothatha mhlambe ihora nohhafu kuya emahoreni amabili futhi neziphuzo ziyotholaka ekupheleni kwengxoxo.

Siqeda kubuyekeza iphepha lemvumo, elichaza ucwaningo kabanzi futhi lusinika imvume yokukhuluma nawe. Awubekelwe ukuthi uphendule yonke imibuzo yami, futhi ungeqa noma imuphi umbuzo. Njengesikhumbuzo, sizosebenzisa isiqophamazwi ukuqopha ingxoxo yethu.

Ikhona imibuzo onayo ngaphambi kokuba siqale ingxoxo?

[Turn on digital recorder.]

I am (MODERATOR NAME) interviewing (FOCUS GROUP ID#) on [DATE] [START TIME]

	Main question/*Imibuzo*	Probe/*Buzisisa*
**Family planning knowledge, attitudes and practices**<br>***Ulwazi ngokuhlela umndeni, indlela abazizwa ngayo, nemikhuba***		
1.1	Please describe your understanding of family planning (or contraception) services.<br><br>*Ngicela ningichazele ukuqonda kwenu ngezinsiza zokuhlela umndeni (noma ukuvikela ukukhulelwa)*	a. Describe the different family planning/contraceptive methods you know about? *Probe for different methods.*<br>b. In your opinion, how well or poorly do family planning/contraceptive methods work to prevent pregnancy?<br>c. Who do you think should use family planning/contraceptive services? *Probe for marital status, parity, age (including teenagers), etc.*<br>d. What do women and girls <u>like</u> about using family planning/contraceptives? (Apart from preventing pregnancy, do they have other positive effects?) *Probe for specific advantages of different methods.*<br>e. What do women and girls <u>not like</u> about using family planning/contraceptives? Why? *Probe for specific things they don't like about different methods?*<br><br>*Explore issues related to gender and perceived benefits/disadvantages.*<br><br>f. When choosing their family planning/contraceptive method, do people think about whether it prevents STIs/HIV?<br><br>a. Chaza ngezinhlobo ezahlukene zokuhlela umndeni/ukuvikela ukukhulelwa owaziyo ngazo? *Buzisisela izindlela ezahlukene.*<br>b. Ngokwenu ukubona, izindlela zokuhlela umndeni/ukuvikela ukukhulelwa zikuvikela kahle noma kabi kanjani ukukhulelwa?<br>c. Nicabanga ukuthi ubani okumele basebenzise izinsiza zokuhlela umndeni/ukuvikela ukukhulelwa? *Buzisisela isimo somshado, isilinganiso, ubudala (kubalwa nentsha), njll.*<br>d. Yini abesifazane Kanye namantombazane <u>abakuthandayo</u> ngokusebenzisa ukuhlela umndeni/ukuvikela ukukhulelwa? (Ngaphandle kokuvimbela ukukhulelwa, banayo eminye imiphumela emihle?) Buzisisa ngobuhle obuthize bezindlela ezahlukene?<br>e. Yini abesifazane namantombazane <u>abangakuthandi</u> ngokusebenzisa ukuhlela umndeni/ukuvikela ukukhulelwa? Kungani? Buzisisa ngezinto ezithize abangazithandi ngezindlela ezahlukene?<br><br>*Hlola izindaba ezihlobene nobulili Kanye zezinzuzo esezibonakele/ububi bayo.*

		f. Uma abantu bekhetha indlela yokuhlela umndeni/ukuvikela ukukhulelwa, ingabe bayacabanga ukuthi iyasivikela isifo esithathelana ngocansi/isandulela ngculaza.
1.2	Do you provide contraceptive and family planning services in your workplace?<br><br>*Ingabe niyazinikezela izinsiza zokuvikela ukukhulelwa kanye nokuhlela umndeni emsebenzini yenu?*	*Yes/No.*<br><br>*Explore what services are available.*<br><br>*a.* Please describe your responsibilities and day-to-day work.<br>*b.* What role do you play in providing contraceptive/family planning services?<br>c. Please describe your family planning and contraceptive counselling procedures.<br>d. Do your facilities have a separate section for providing family planning/contraceptive services? Or is this service offered across all sections?<br>e. How often do women change their family planning/contraceptive methods used? *Explore why and how this is done?*<br><br>*Yebo/Cha.*<br><br>*Hlola yiziphi izinsiza ezitholakalayo.*<br><br>*a.* Ngicela ningichazela ngenikwenzayo kanye nemisebenzi yenu yosuku nosuku.<br>*b.* Iyiphi indima eniyidlalayo ekunikezeleni izinsiza zokuvikela ukukhulelwa/ukuhlela umndeni?<br>c. Ngicela nichaze izinqubo zenu (counselling) zokuhlela umndeni Kanye nokweluleka ngokuvikela ukukhulelwa.<br>d. Ingabe izikhungo zenu zinayo ingxenye eseceleni yokunikezela izinsiza zokuhlela umndeni/ukuvikela ukukhulelwa? Noma lolusizo nilunikezelwa kuzozonke izikhungo/izingxenye?<br>e. Bavamise ukuzishintsha kanganani abesifazane izindlela zabo zokuhlela umndeni/ukuvikela ukukhulelwa ezisetshenziswayo? Hlola kungani futhi kwenziwa kanjani lokhu?
1.3	What does your community know about family planning and contraceptive methods?<br><br>*Yini eyaziwa umphakathi wenu ngezindlela zokuhlela umndeni kanye nokuvikela ukukhulelwa?*	a. What is/are the most common method(s) of family planning/contraception used in your community?<br>b. Why do you think this is/are the most common method(s)?<br><br>a. Yini/yiziphi izindlela ezijwayeleke kakhulu zokuhlela umndeni/ukuvikela ukukhulelwa ezisetshenziswa emphakathini wenu?<br>b. Nicabanga ukuthi kungani kuyilezindlela ezijwayeleke kakhulu?

1.4	What family planning/contraceptive methods are available in your community?<br><br>*Iziphi izindlela zokuhlela umndeni/ukuvimbela ukukhulelwa ezitholakalayo emphakathini wakho?*	a. What things make it difficult for young women to get and use family planning/contraceptive methods to prevent pregnancy when they want to use them?<br><br>*Probes: things about health services and health workers; other people's opinions about young women using family planning/contraception (especially teenagers and unmarried women); whether or not people already have children; male partners' opinions, etc.*<br><br>b. Are family planning/contraceptive services freely available at your facilities?<br><br>a. Iziphi izinto ezenza kubenzima kwabesifazane ukuthola nokusebenzisa izinsiza zokuhlela umndeni/ukuvikela ukukhulelwa ukuvimbela ukukhulelwa mabefuna ukuzisebenzisa?<br><br>*Buzisisa: izinto ngezinsiza zempilo nabasebenzi bezempilo; imibono yabantu ngabantu besifazane abasebancane abasebenzisa izindlela zokuhlela umndeni/ukuvikela ukukhulelwa (ikakhulukazi abantu abasebasha, Kanye nabashadile) asebevele benazo noma abanazo izingane; imibono yophathina besilisa, njll.*<br><br>b. Ingabe izinsiza zokuhlela umndeni/ukuvikela ukukhulelwa zitholakala mahhala emtholampilo wenu?
1.5	How do women access family planning and contraceptive services in your community?<br><br>*Abantu besifazane bafinyelela kanjani ezinsizeni zokuhlela umndeni kanye nokuvikela ukukhulelwa emphakathini yenu?*	*Explore where, how they get the method, travel requirements, who they get them from?*<br><br>*Give each participant a paper and a pen and ask them:*<br>Please draw a map of where family planning and contraceptive services are available in your communities.<br><br>*Hlola kuphi, bayithola kanjani indlela (method), izidingo zokuhamba, bazithola kubani?*<br><br>*Nikeza ababambiqhaza ngamunye ipeni nephepha futhi ubacele:*<br>Ngicela nidwebe ibalazwe (map) lapho kutholakala khona izinsiza zokuhlela umndeni kanye nokuvikela ukukhulelwa emphakathini yenu.
1.6	Who are the most important people in supporting women and girls in choosing and using family planning and contraceptive methods?	*Probe for*<br>• Partner<br>• Friends<br>• Parents<br>• Health workers<br>• Community leaders<br>• Religious leaders<br><br>*Explore why these people are the most important.*

	Obani abantu ababaluleke khakhulu ekusizeni abasifazane Kanye namantombazane ekukhetheni Kanye nasekusebenziseni izindlela zokuhlela umndeni kanye nokuvikela ukukhulelwa?	*Buzisisela*<br>• Uphathina<br>• Abangani<br>• Abazali<br>• Abasebenzi bezempilo<br>• Abaholi bomphakathi<br>• Abaholi bezenkolo<br><br>*Chaza kungani bebaluleke kakhulu labantu.*
1.7	How comfortable are you counselling about family planning methods?<br><br>*Nikhululeke kangakanani ekwelulekeni ngezindlela zokuhlela umndeni?*	a. Why?<br>b. Who are you most comfortable to counsel? *Probe for age, sex, marital status, etc.*<br><br>a. Kungani?<br>*b.* Obani enikhululeke kakhulu ukubeluleka? Buzisisela iminyaka, ubulili, isimo somshado, njll.
**Health services capacity**<br>***Amandla ezinsiza zezempilo***		
2.1	What is the capacity of your facilities to provide family planning/contraceptive services?<br><br>*Angakanani amandla (capacity) ezikhungo zenu ekunikezeleni izinsiza zokuhlela umndeni/ukuvikela ukukhulelwa?*	a. Do they have sufficient resources?<br>b. Please explain who is on staff at your facilities: how many doctors, nurses, others?<br>c. On an average day, how many clients are seen at the facilities?<br>d. How many clients come to your facilities for family planning and contraceptive services?<br>e. What community/communities are served by your health facilities?<br>f. What is the average time of a routine client visit for family planning/contraceptive services?<br><br>a. Ingabe zinezinsiza (Resources) ezanele?<br>b. Ngicela nichaze ukuthi obani abakubasebenzi esikhungweni senu: bangaki odokotela, abahlengikazi, nabanye?<br>c. Osukwini nje olujwayelekile, zingaki iziguli ezibonwayo ezikhungweni?<br>d. Zingaki iziguli eziza ezikhungweni zenu ngezinsiza zokuhlela umndeni kanye nokuvikela ukukhulelwa?<br>e. Yimuphi/yimiphi imiphakathi enikezwa usizo izikhungo zenu zezempilo?<br>f. Kulinganiselwa esikhathini esingakanani ukuvakasha okwejwayelekile ngezinsiza zokuhlela umndeni/nokuvikela ukhulelwa?
2.2	What role do you think healthcare providers play in assisting young people to access contraceptive/family planning methods?	a. What role do you think they should play?<br>b. Do you think that the family planning/contraceptive needs of the young people are met by the healthcare providers?<br>c. Do you think that healthcare providers and young people have the same goals/vision for providing and accessing contraceptive/family planning services?

	Iyiphi indima enicabanga ukuthi abanikezeli bonakekelo lwezempilo bayayidlala ekusizeni abantu abancane ekufinyeleleni kwizindleleni zokuvikela ukukhulelwa/ukuhlela umndeni?	a. Nicabanga ukuthi iyiphi indima okumele bayidlale?<br>b. Nicabanga ukuthi izidingo zokuhlela umndeni/ukuvikela ukukhulelwa zabantu abasebancane ziyafezwa ngabanikeli bonakekelo lwezempilo?<br>c. Nicabanga ukuthi abanikezeli bonakekelo lwezempilo banempokophelo (goal)/umbono ofanayo yokunikezelwa kanye nokufinyelela ezinsizeni zokuvikela ukukhulelwa/ukuhlela umndeni.
2.3	Who are the major clients in your community who access family planning/contraceptive services?<br><br>*Obani abantu okuyibonabona emphakathini yenu abafinyelela ezinsizeni zokuhlela umndeni/ukuvikela ukukhulelwa?*	*Explore categories such as age, sex, marital status, rural vs urban, etc.*<br><br>*Hlola izinhlobo (categories) ezifana nobudala, ubulili, isimo somshado, amakhaya uqhathanisa nedolobha, njll.*
2.4	What barriers and enablers are there to providing family planning and contraceptive services in your health facilities?<br><br>*Yini okuvimbelayo Kanye nokuvumelayo (enablers) okukhona ekunikezeleni izinsiza zokuhlela umndeni Kanye nokuvikela ukukhulelwa ezikhungweni zenu zezempilo?*	*Explore barriers and enablers.*<br>*Probe on available resources, number of staff, operation hours, number of rooms available vs number of clients attending the facility, waiting time, etc.*<br><br>*Hlola okuvimbelayo Kanye nokuvumelayo*<br>*Buzisisa ezinsizeni ezitholakalayo, inani lababsebenzi, izikhathi zokusebenza, inani lezindlu ezitholakalayo liqhathaniswa nenani labantu abaze esikhungweni, isikhathi sokulinda, njll.*
2.5	Are there any religious or cultural barriers or facilitators to accessing family planning/contraceptive services?<br><br>*Ingabe kukhona ukuvimbela kwenkolo noma kosiko noma okwenza kubelula ukufinyelela ezinsizeni zokuhlela umndeni/ukuvikela ukukhulelwa?*	*Explore barriers and facilitators at both religious and cultural level.*<br><br>*a. If barriers were reported:*<br>How can these be overcome?<br><br>*Hlola okuvimbelayo nokwenza kubelula kukho kokubili ezingeni lenkolo Kanye nokosiko.*<br><br>*a. Uma ukuvimbela kwakubikiwe:*<br>Kunganqotshwa kanjani lokhu?
2.6	How do you think decentralisation of services has affected (or could affect) community access to contraceptive/ family planning services?<br><br>*Nicabanga ukuthi ukusabalaliswa kwezinsiza kukuthinte (noma kungakuthinta) kanjani ukufinyelela komphakathi ezinsizeni zokuvikela ukukhulelwa/ukuhlela umndeni?*	*Explore both positive and negative outcomes.*<br><br>*Hlola yomibili emihle nemibi imiphumela.*

2.7	Are there any special services for women and girls wanting family planning and contraceptive services at your health facilities or in the local community?<br><br>*Zikhona izinsiza ezikhethelekile zabesifazane Kanye namantombazane abafuna izinsiza zokuhlela umndeni umndeni Kanye nokuvikela ukukhulelwa ezikhungweni zenu zezempilo noma emphakathini wendawo?*	*Explore what these services are, and where they are.*<br>*If there are services, explore their accessibility and whether they are used or not.*<br><br>*Hlola yiziphi lezinsiza, futhi zikuphi.*<br>*Uma kunezinsiza, hlola ukufinyeleleka kuzo Kanye nokuthi ziyasetshenziswa noma cha.*
2.8	What, if any, unique needs do women and girls in your community have in the context of accessing family planning and contraceptive services?<br><br>*Yiziphi, uma zikhona, izidingo ezehlukile abesifazane Kanye namantombazane emphakathini wenu abanazo endabeni (issue) yokufinyelela ezinsizeni zokuhlela umndeni Kanye nokuvikela ukukhulelwa?*	
**Quality of care**<br>***Izinga lonakekelo***<br>***Note to facilitator: Remember to ask about quality (and not level) of care***		
3.1	How would you define good quality family planning/contraceptive services?<br><br>*Ungalichaza kanjani unakekelo oluhle lwezinsiza zokuhlela umndeni/nokuvikela ukukhulelwa?*	What constitutes good quality of care?<br><br>Yini eyakha unakekelo oluhle?
3.2	Some people say quality of care is influenced by issues such as available health care workers, integration of services, facility operation hours, number of rooms available, number of clients attending the facility, waiting time, etc. Which of these are important for you in describing good quality care?<br><br>*Abanye abantu bathi izinga lonakekelo lithelelwa izindaba ezifana nabasebenzi bonakekelo lwezempilo abatholakalayo, ukuhlanganiswa kwezinsiza, izikhazi zokusebenza zesikhungo, inani lezundlu ezitholakalayo, inani leziguli ezifikayo esikhungweni, isikhathi sokulinda, njll. Yikuphi kulokhu okuyinona okubalulekile kinina ekuchazeni izinga elihle lonakekelo?*	

3.3	Are quality family planning/contraceptive services available to people in your community?<br><br>*Ingabe izinsiza ezisezingeni elihle zokuhlela umndeni/ukuvikela ukukhulelwa ziyatholakala kubantu emphakathini wakho?*	*Yes/No.*<br><br>*a.* Are your health care facilities providing good quality family planning/contraceptive services? *Yes/No.*<br><br>*Explore using next question.*<br><br>*Yebo/Cha.*<br><br>*b.* Ingabe izikhungo zenu zinikezela izinsiza ezisezingeni elihle zokuhlela umndeni/ukuvikela ukukhulelwa? Yebo/cha.<br><br>*Hlola usebenzisa umbuzo olandelayo.*
3.4	How could your facilities provide better quality family planning/contraceptive services?<br><br>*Zinganikezela kanjani izikhungo zenu izinsiza ezisezingeni elincono lokuhlela umndeni/ukuvikela ukukhulelwa?*	a. How do you think the services should be delivered?<br>b. Who should deliver the services?<br>c. What other information should be given to clients about family planning/contraceptive services?<br><br>a. Nicabanga ukuthi kumele zilethwe kanjani izinsiza?<br>b. Ubani okumele alethe lezinsiza?<br>c. Yiluphi olunye ulwazi okumele lunikezwe kubantu ngezinsiza zokuhlela umndeni/ukuvikela ukukhulelwa?
**Community participation and relationships**<br>***Iqhaza lomphakathi kanye nobudlelwano***		
4.1	Community members and groups participate in different ways within the health system. How would you define community participation in this community?<br><br>*Amalunga omphakathi namaqembu abambiqhaza ngezindlela ezahlukene phakathi ohlelweni lwezempilo. Ungakuchaza kanjani ukubamba iqhaza komphakathi kulomphakathi?*	*Explore group understanding vs individual opinions?*<br><br>*Hlola ukuqonda kweqembu uqhathanisa namuntu ngamunye?*
4.2	What are some of the existing community participation activities in this area?	*a.* Who participates in these activities? And how?<br>b. How does the community feel about these activities?<br>c. What community participation activities work and which ones don't work? *Explore why– probe for issues of age, religion and cultural acceptability of community participation.*<br>d. What are some of the challenges to community participation in your area? *Also explore if no community participation activities in the area.*<br>e. Who should participate if a project is created on family planning and contraceptive service in this community? How should they participate?

	Imiphi eminye imisebenzi ekhona umphakathi ebambe kuyo iqhaza kulendawo?	a. Ubani obamba iqhaza kulemisebenzi? Futhi kanjani?<br>b. Umphakathi uzizwa kanjani ngalemisebenzi?<br>c. Imiphi imsebenzi esebenzayo nengasebenzi umphakathi obamba kuyo iqhaza? Hlola kungani-buzisisa ezindabeni ezifana nobudala, inkolo kanye nokwamukeleka ngokosiko kokubamba iqhaza komphakathi.<br>d. Iziphi ezinye zezinselelo zokubamba iqhaza komphakathi endaweni yakho? *(Phinda uhlole ukuthi ayikho yini imisenzi umphakathi obamba kuyo iqhaza endaweni).*<br>e. Ubani okumele abambe iqhaza emphakathini uma iprojethi yakhiwe ezinsizeni zokuhlela umndeni Kanye nokuvikela ukukhulelwa kulomphakathi? Kumele balibambe kanjani iqhaza?
4.3	How do you engage with the community about family planning/contraceptive services?<br><br>*Nihlanganyela kanjani nomphakathi ngezinsiza zokuhlela umndeni/ukuvikela ukukhulelwa?*	a. How do you, as health care providers, feel about engaging with the community about family planning/contraceptive services?<br><br>a. Nizizwa kanjani nina njengabanikezeli bonakekelo lwezempilo ngokuhlanganyela nomphakathi mayelana nezinsiza zokuhlela umndeni/ukuvikela ukukhulelwa?
4.4	How do health care providers take community voices about family planning/contraceptive services into account?<br><br>*Abanikezeli bonakekelo lwezempilo baluthatha kanjani balunake (take into account) uvo lomphakathi ngezinsiza zokuhlela umndeni/ukuvikela ukukhulelwa?*	*Explore.*<br><br>a. Who initiates discussions on family planning/contraception? (Clients vs health care providers)<br><br>*Hlola.*<br><br>a. Ubani oqala izingxoxo zokuhlela/ukuvikela ukukhulelwa? (Iziguli uqhathanisa nabanikezeli bonakekelo lwezempilo)
4.5	Do you have links with other health care providers in the community?<br><br>*Ninalo uxhumano (links) nabanye abanikezeli bonakekelo lwezempilo emphakathini?*	Describe these relations/links.<br><br>Chaza lokhuzwana/ukuxhumana.
4.6	How do you think community participation can be used to improve access to family planning/contraceptive services?	What are your recommendations for improving community engagement with health care providers when accessing family planning/contraceptive services?<br><br>*Probe for consideration of age (teenagers vs older women), married vs unmarried, rural vs urban, women with or without children, etc.*

	Ucabanga ukuthi ukubamba iqhaza komphakathi kungasetshenziswa kanjani ekwenzeni ncono ukufinyelela ezinsizeni zokuhlela umndeni/ukuvikela ukukhulelwa?	Yikuphi ongakuncoma ekuthuthukiseni ukuzibandakanya komphakathi nabanikezeli bonakekelo lwezempilo ekufinyeleleni ezinsizeni zokuhlela umndeni/nokuvikela ukukhulelwa?<br><br>*Buzisisa ngokucabangela ubudala (intsha iqhathaniswa nabesifazane abadala, abashadile beqhathaniswa nabangashadile, amakhaya eqhathaniswa nedolobha, abesifazane abanabo noma abangenabo abantwana nokunye)*
4.7	What role do you think the community should play to improve future access to family planning/contraceptive services?<br><br>*Ucabanga ukuthi iyiphi indima umphakathi okumele uyidlale ukuthuthukisa ukufinyelela okuzayo ezinsizeni zokuhlela umndeni/ukuvikela ukukhulelwa?*	a. How can the community be engaged in future interventions for improved uptake of family planning/contraceptive services?<br>b. What could these interventions be?<br>c. *Explore.*<br><br>a. Umphakathi ungabandakanyeka kanjani ekungeneleleni okuzayo bokuthuthukisa izinsiza ze-uptake yokuhlela umndeni/ukuvikela ukukhulelwa?<br>b. Kungabe yikuphi lokhukungenelela?<br>c. *Hlola.*
**Conclusion**<br>***Isiphetho***		
5.1	Do you have anything else that you would like to tell us about family planning/contraception and community participation before we end?<br><br>*Kukhona okunye onakho ongathanda ukusitshela khona mayelana nokuhlela umndeni/ukuvikela ukukhulelwa Kanye nokubamba iqhaza komphakathi ngaphambi kokuba siqede?*	

This is the end of our discussion. Thank you for your time.
Sekuyisiphetho sengxoxo yethu lesi. Ngiyabonga ngesikhathi sakho.
