# Supplementary material for: Access to public sector family planning services and modern contraceptive methods in South Africa: A qualitative evaluation from community and health care provider perspectives
Source: PLoS One. 2023 Mar 17;18(3):e0282996. doi: 10.1371/journal.pone.0282996 (PMC10022780; doi:10.1371/journal.pone.0282996)
Supplement: S1 Table — (DOCX) [file pone.0282996.s006.docx]

***Supporting Information 1 - Data from the community participants’ drawings.***

| **Group** | **Mean age of group (years)** | **Modes of transport** | **Average travel time (minutes)** | **Sources of FP/C** |
| --- | --- | --- | --- | --- |
| Female FGD urban adults (n=8) | 41.8 | Walking only (n=8) | Mean = 13  Range = [5;15] | Local PHC only |
| Female FGD urban young adults (n=8) | 23.1 | Walking only (n=7)  Walking and taxi (n=) | Mean = 29  Range = [10;60] | Local PHC only |
| Female FGD urban adolescents (n=9) | 17.9 | Walking only (n=7)  Public taxi only (n=1)  Walking and taxi (n=1) | Mean = 18  Range = [10;60] | Local PHC only |
| Female FGD rural adults (n=7) | 38 | Walking only (n=7) | Mean = 44  Range = [5; 60] | Local PHC only |
| Female FGD rural young adults (n=10) | 25 | Walking only (n=10) | Mean = 51  Range = [5; 120] | Local PHC only |
| Female FGD rural adolescents (n=10) | 16.7 | Walking only (n=10) | Mean = 66  Range = [30; 120] | Local PHC only |
| Female FGD women in union (n=10) | 30.7 | Walking only (n=6)  Walking and taxi (n=3) | Mean = 21  Range = [10; 30] | Local PHC only |
| Female FGD unmarried/single women (n=8) | 26.6 | Walking only (n=6)  Walking and taxi (n=1) | Mean = 45  Range = [13; 60] | Local PHC only |
| Female FGD women without children (n=8) | 22 | Walking only (n=7)  Public taxi only (n=1) | Mean = 26  Range = [10; 45] | Local PHC only |
| Male FGD adults (n=7) | 37.6 | Walking only (n=5)  Public taxi only (n=1)  Walking and taxi (n=1) | Mean = 31  Range = [5; 50]  Shop travel time  Mean = 9.2  Range [5;30] | Local PHC only (n=2)  Local PHC and shop (n=5) |
| Male FGD young adults (n=8) | 21.3 | Walking only (n=8) | Mean = 28  Range = [10; 45]  Shop travel time  Mean = 17.5  Range = [5;30] | Local PHC only (n=1)  Local PHC and shop (n=7) |
| Male FGD adolescents (n=10) | 16.4 | Walking only (n=10) | Mean = 86 minutes  Range = [5;240] | Local PHC only (n=9)  Shop only (n=1) |
