# Supplementary figures and images for: Access to public sector family planning services and modern contraceptive methods in South Africa: A qualitative evaluation from community and health care provider perspectives
Source: PLoS One. 2023 Mar 17;18(3):e0282996. doi: 10.1371/journal.pone.0282996 (PMC10022780; doi:10.1371/journal.pone.0282996)

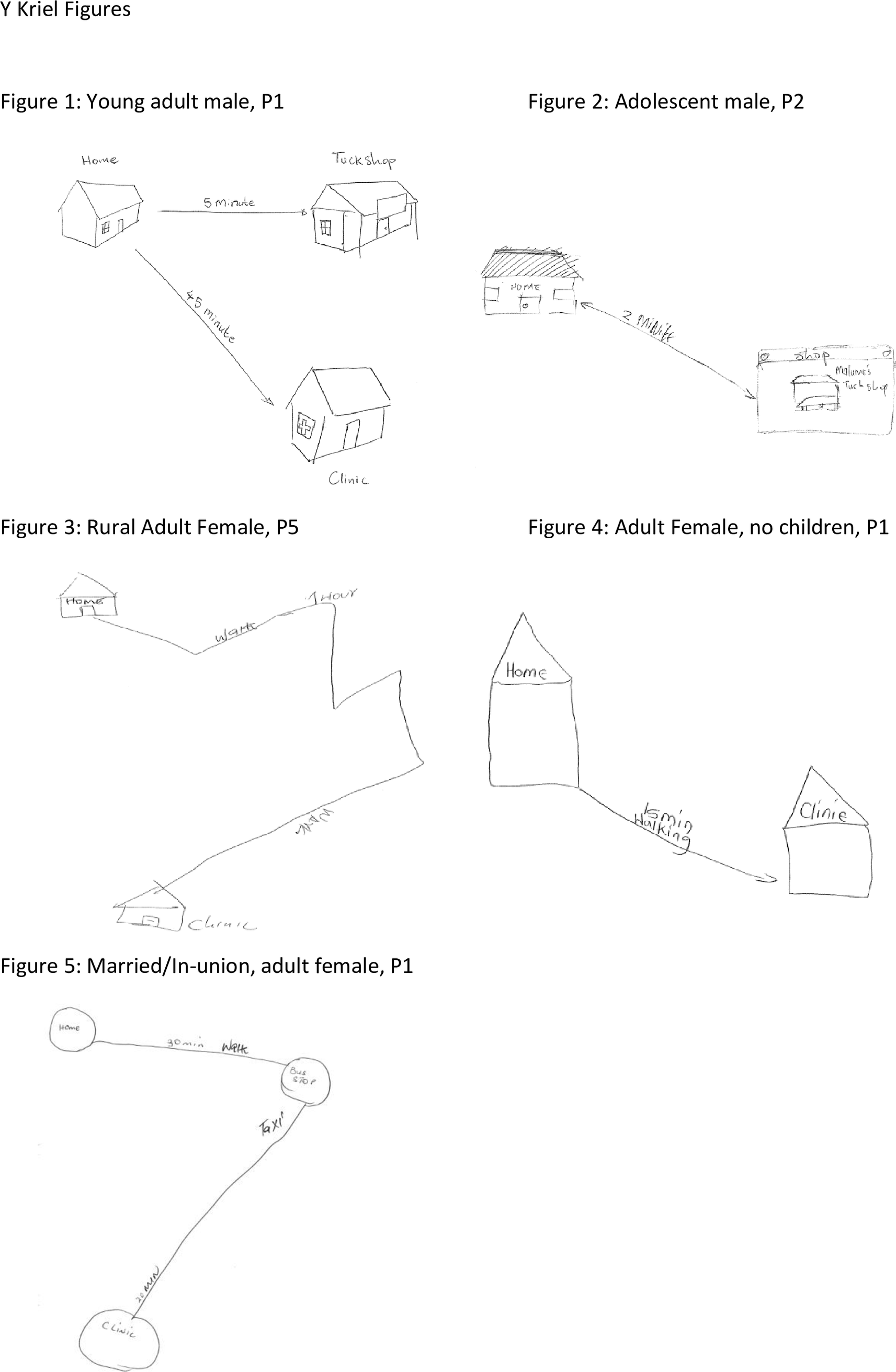

Supplement: S1 Fig — (TIF) [file pone.0282996.s007.tif]
