# Supplementary material for: Access to public sector family planning services and modern contraceptive methods in South Africa: A qualitative evaluation from community and health care provider perspectives
Source: PLoS One. 2023 Mar 17;18(3):e0282996. doi: 10.1371/journal.pone.0282996 (PMC10022780; doi:10.1371/journal.pone.0282996)
Supplement: Attachment — Submitted filename: Track changes_PONE-D-21-18604_reviewer.pdf [file pone.0282996.s008.pdf]

PLOS ONE

Access to public sector family planning services and modern contraceptive methods in South Africa: A qualitative evaluation from community and health care provider perspectives.

--Manuscript Draft--

**Manuscript Number:**	PONE-D-21-18604
**Article Type:**	Research Article
**Full Title:**	Access to public sector family planning services and modern contraceptive methods in South Africa: A qualitative evaluation from community and health care provider perspectives.
**Short Title:**	Access to modern contraception in South Africa: A qualitative study
**Corresponding Author:**	Yolandie Kriel, MSc<br>University of the Witwatersrand Maternal Adolescent and Child Health<br>Durban, KwaZulu Natal SOUTH AFRICA
**Keywords:**	family planning; contraception; Access; South Africa; community; health care provider; Qualitative research
**Abstract:**	Progress has been made to improve access to family planning services and contraceptive methods, yet many women still struggle to access contraception which increases their risk for unintended pregnancy. This is also true for South Africa where over fifty percent of pregnancies are reported as unintended, even though contraception is freely available. There is also a stagnation in fertility reate indicators and contraceptive use data, indicating that there may be challenges to accessing contraception. This paper explores the evaluation of access to contraception from community and health care provider perspectives.<br>In this qualitative study factors affecting the uptake and use of contraception was explored through focus group discussions, in-depth interviews and drawings. Particiaptns included male and female community members between 15 and 49 years of age, health care providers, and key stakeholder informants. A thematic content analysis approach was used to explore the data using NVivo 10. Emergent themes were elucidated and thematically categorised.<br>The results were categorised according to a priori access components. Overall, community members reported being satisfied with the accessibility and affordability components, less satisfied with availability of a variety of contraceptive methods and providers, and least satisfied with accommodation. Data from the illustrations showed that adolescent males experienced the most geopgraphic barriers. Male partners reported that they had to provide financial resources to their female partners to obtain contraception. Participants also reported limited choice in the source and methods of contraception available to them. The effects of integrated care, such as long waiting times, and rude health care providers were cited as reasons to discontinue contraceptive use, which increases the risk of unintended pregnancy. The data illustrated the importance of examining access as a holistic concept and not just the availability of services or methods.
**Order of Authors:**	Yolandie Kriel, MSc
	Cecilia Milford
	Joanna Paula Cordero
	Fatima Suleman
	Petrus S Steyn
	Jennifer Ann Smith
**Additional Information:**	
**Question**	**Response**
**Financial Disclosure**	Authors: PSS, JPC, JAS

Enter a financial disclosure statement that describes the sources of funding for the work included in this submission. Review the submission guidelines for detailed requirements. View published research articles from *PLOS ONE* for specific examples.<br><br>This statement is required for submission and **will appear in the published article** if the submission is accepted. Please make sure it is accurate.<br><br>**Unfunded studies**<br>Enter: *The author(s) received no specific funding for this work.*<br><br>**Funded studies**<br>Enter a statement with the following details:<br>• Initials of the authors who received each award<br>• Grant numbers awarded to each author<br>• The full name of each funder<br>• URL of each funder website<br>• Did the sponsors or funders play any role in the study design, data collection and analysis, decision to publish, or preparation of the manuscript?<br>• **NO** - Include this sentence at the end of your statement: *The funders had no role in study design, data collection and analysis, decision to publish, or preparation of the manuscript.*<br>• **YES** - Specify the role(s) played.<br><br>* typeset	This work received funding through a grant received by UNDP-UNFPA-UNICEF-WHO-World Bank Special Programme of Research, Development and Research Training in Human Reproduction (HRP), a cosponsored programme executed by the World Health Organization (WHO) from the Bill and Melinda Gates Foundation [OPP1084560] and the United States Agency for International Development (USAID) through the USAID/WHO Umbrella Grant 2016-2018.<br><br>No - The funders had no role in the study design, data collection and analysis, decision to publish, or preparation of the manuscript.
**Competing Interests**<br><br>Use the instructions below to enter a competing interest statement for this submission. On behalf of all authors, disclose any competing interests that could be perceived to bias this work—acknowledging all financial support and any other relevant financial or non-financial competing interests.<br><br>This statement is required for submission and **will appear in the published article** if	The authors have declared that no competing interests exists.

the submission is accepted. Please make sure it is accurate and that any funding sources listed in your Funding Information later in the submission form are also declared in your Financial Disclosure statement.<br><br>View published research articles from *PLOS ONE* for specific examples.<br><br>**NO authors have competing interests**<br><br>Enter: *The authors have declared that no competing interests exist.*<br><br>**Authors with competing interests**<br><br>Enter competing interest details beginning with this statement:<br><br>*I have read the journal's policy and the authors of this manuscript have the following competing interests: [insert competing interests here]*<br><br>* typeset	
**Ethics Statement**<br><br>Enter an ethics statement for this submission. This statement is required if the study involved:<br><br>• Human participants<br>• Human specimens or tissue<br>• Vertebrate animals or cephalopods<br>• Vertebrate embryos or tissues<br>• Field research<br><br>Write "N/A" if the submission does not require an ethics statement.<br><br>General guidance is provided below. Consult the submission guidelines for detailed instructions. **Make sure that all information entered here is included in the Methods section of the manuscript.**	This study received World Health Organization (WHO) Ethics Review Committee (ERC) (Project ID A65896) and Research Project Review Panel (RP2) approval. Local country ethics review and approval was provided by the University of the Witwatersrand Human Research Ethics Committee (Health- HREC, reference no 1504101). Further ethics reciprocity was provided by the University of KwaZulu-Natal's Biomedical Research Ethics Committee (BREC). Permission was also obtained from the KwaZulu-Natal Provincial Department of Health to interview health care providers. All participants voluntarily signed an informed consent form, which included permission to audio record the interview/group sessions. Consent for minors (those aged <18years) was obtained by their identified parents or legal guardians. Assent was obtained from the minors themselves to participate.

**Format for specific study types**<br><br>**Human Subject Research (involving human participants and/or tissue)**<br>• Give the name of the institutional review board or ethics committee that approved the study<br>• Include the approval number and/or a statement indicating approval of this research<br>• Indicate the form of consent obtained (written/oral) or the reason that consent was not obtained (e.g. the data were analyzed anonymously)<br><br>**Animal Research (involving vertebrate animals, embryos or tissues)**<br>• Provide the name of the Institutional Animal Care and Use Committee (IACUC) or other relevant ethics board that reviewed the study protocol, and indicate whether they approved this research or granted a formal waiver of ethical approval<br>• Include an approval number if one was obtained<br>• If the study involved *non-human primates*, add *additional details* about animal welfare and steps taken to ameliorate suffering<br>• If anesthesia, euthanasia, or any kind of animal sacrifice is part of the study, include briefly which substances and/or methods were applied<br><br>**Field Research**<br><br>Include the following details if this study involves the collection of plant, animal, or other materials from a natural setting:<br>• Field permit number<br>• Name of the institution or relevant body that granted permission	
**Data Availability**<br><br>Authors are required to make all data underlying the findings described fully available, without restriction, and from the time of publication. PLOS allows rare exceptions to address legal and ethical concerns. See the PLOS Data Policy and FAQ for detailed information.	No - some restrictions will apply

A Data Availability Statement describing where the data can be found is required at submission. Your answers to this question constitute the Data Availability Statement and **will be published in the article**, if accepted.<br><br>**Important:** Stating 'data available on request from the author' is not sufficient. If your data are only available upon request, select 'No' for the first question and explain your exceptional situation in the text box.<br><br>Do the authors confirm that all data underlying the findings described in their manuscript are fully available without restriction?	
**Describe where the data may be found in full sentences. If you are copying our sample text, replace any instances of XXX with the appropriate details.**<br><br>• If the data are **held or will be held in a public repository**, include URLs, accession numbers or DOIs. If this information will only be available after acceptance, indicate this by ticking the box below. For example: *All XXX files are available from the XXX database (accession number(s) XXX, XXX.).*<br>• If the data are all contained **within the manuscript and/or Supporting Information files**, enter the following: *All relevant data are within the manuscript and its Supporting Information files.*<br>• If neither of these applies but you are able to provide **details of access elsewhere**, with or without limitations, please do so. For example:<br><br>*Data cannot be shared publicly because of [XXX]. Data are available from the XXX Institutional Data Access / Ethics Committee (contact via XXX) for researchers who meet the criteria for access to confidential data.*<br><br>*The data underlying the results presented in the study are available from (include the name of the third party*	The database is not publicly available as it contains information that could compromise research participant privacy/consent. However, some anonymised aspects of the datasets may be available upon request and with permission of the Department of Reproductive Health and Research, World Health Organisation. Note that data sharing is subject to WHO data sharing policies and data use agreements with the participating research centres.

and contact information or URL).<br>• This text is appropriate if the data are owned by a third party and authors do not have permission to share the data.<br><br>* typeset	
Additional data availability information:	Tick here if your circumstances are not covered by the questions above and you need the journal's help to make your data available.

Powered by Editorial Manager® and ProduXion Manager® from Aries Systems Corporation

Manuscript

Click here to access/download;Manuscript;Y Kriel Paper 3 Main Manuscript 31May21.docx

Access to public sector family planning services and modern contraceptive methods in South Africa: A qualitative evaluation from community and health care provider perspectives.

Authors: Yolandie Kriel RN MHSc[1,2*], Cecilia Milford PhD[1], Joanna Paula Cordero MA[4], Fatima Suleman PhD[3], Petrus S Steyn MD, MPhil[4], Jennifer Ann Smit PhD[1].

Author Affiliations:

[1]MRU (MatCH Research Unit), Department of Obstetrics and Gynaecology, Faculty of Health Sciences, University of the Witwatersrand, Durban, South Africa

[2]School of Public Health and Nursing, College of Health Sciences, University of KwaZulu-Natal, Durban, South Africa

[3]Discipline of Pharmaceutical Science, College of Health Sciences, University of KwaZulu-Natal, Westville, South Africa

[4]UNDP-UNFPA-UNICEF-WHO-World Bank Special Programme of Research, Development and Research Training in Human Reproduction (HRP), Department of Sexual and Reproductive Health and Research, World Health Organization, Geneva, Switzerland.

Corresponding author*

Ms. Yolandie Kriel RN MHSc, MRU (MatCH Research Unit), Department of Obstetrics and Gynaecology, Faculty of Health Sciences, University of the Witwatersrand, Durban, South Africa.

Email: ykriel@mru.ac.za

Phone: +27310011917

Abstract

Progress has been made to improve access to family planning services and contraceptive methods, yet many women still struggle to access contraception which increases their risk for unintended pregnancy. This is also true for South Africa where over fifty percent of pregnancies are reported as unintended, even though contraception is freely available. There is also a stagnation in fertility reate indicators and contraceptive use data, indicating that there may be challenges to accessing contraception. This paper explores the evaluation of access to contraception from community and health care provider perspectives.

In this qualitative study factors affecting the uptake and use of contraception was explored through focus group discussions, in-depth interviews and drawings. Particiaptns included male and female community members between 15 and 49 years of age, health care providers, and key stakeholder informants. A thematic content analysis approach was used to explore the data using NVivo 10. Emergent themes were elucidated and thematically categorised.

The results were categorised according to *a priori* access components. Overall, community members reported being satisfied with the accessibility and affordability components, less satisfied with availability of a variety of contraceptive methods and providers, and least satisfied with accommodation. Data from the illustrations showed that adolescent males experienced the most geopgraphic barriers. Male partners reported that they had to provide financial resources to their female partners to obtain contraception. Participants also reported limited choice in the source and methods of contraception available to them. The effects of integrated care, such as long waiting times, and rude health care providers were cited as reasons to discontinue contraceptive use, which increases the risk of unintended pregnancy. The data illustrated the importance of examining access as a holistic concept and not just the availability of services or methods.

Key words:

Family planning, contraception, access, South Africa, community, health care provider

Introduction

Family planning (FP) services and modern contraceptive methods are some of the most important public health strategies that can improve the lives of individuals, families, communities, and nations. Access to FP services and contraception is a Human Right and a target set out by the Sustainable Development Goals (SDG) target 3.7 (1). At the London Summit in 2012, Family Planning 2020 (FP2020) was established with the goal to get an additional 120 million users onto contraception (2). The SDG target, FP2020, along with General Comments No. 14 and No. 22 set out by the United Nations Committee on Economic, Social, and Cultural Rights (3), are driving goals for the international family planning community and nations, including South Africa (SA), to ensure equitable access to FP services and contraceptive methods.

Results from national surveys show that although much progress has been made over the past couple of decades, many women in low-and-middle-income countries (LMICs), including women in SA, struggle to access FP services and contraceptive methods (2, 4, 5). An estimated 230 million women and adolescent girls are in need of modern contraception (2, 6) in LMICs, while Sub-Saharan Africa remains the region with the highest fertility and unmet need for contraception (7-9).

One of the key challenges in studying and evaluating access is that it remains a difficult concept to define. In their seminal work, Penchansky and Thomas (10) proposed the definition of access as “the general concept which summarizes a set of more specific areas of fit between the patient and the health care system” (p.128). They outlined five key independent but interrelated components – accessibility, availability, affordability, accommodation, and acceptability (10). Despite this established framework, numerous other frameworks have been proposed over the last three decades - see, for example, the UNCESCR (United Nations Committee on Economic, Social, and Cultural Rights) availability, accessibility, acceptability, and good quality (AAAQ) framework (11, 12), or the FP focused access frameworks proposed by Choic et al (13), and Bertrand et al (14). A critique of these other access frameworks is the interchangeable use of the independent components.

Bertrand argues that there is little value in separating out the components, yet numerous other studies have demonstrated that the availability of services does not mean they are accessible or obtainable (4, 15). To get a true measure of access, components should be evaluated independently where possible. Another important issue in the study of access is to separate out the concept of access from the concept of quality of care (QoC). Much ambiguity and overlap exist between these two important concepts within the FP literature (13, 14, 16). However, to accurately identify where challenges pose the most significant barriers to accessing FP services, a distinction must be made between access and QoC (16).

Qualitative, exploratory data can provide critical understandings about factors influencing access (13), which can lead to the development of community-focused interventions to improve uptake and met need for modern contraception. There is currently a paucity in the literature about qualitative understandings on access to FP services and contraception. This paper aimed to fill that gap by focusing on qualitative findings from South Africa. This aim was achieved by identifying specific factors related to each access component and their influence on contraceptive uptake and use from a community and health care provider (HCP) perspective.

<u>Background</u>

South Africa has one of the longest-running modern family planning programmes in Sub-Saharan Africa (17). Modern contraception is mainly provided free of charge through the public health sector at primary health care (PHC) clinics that operate from 08:00 am to 16:00 pm on weekdays. Reproductive health challenges such as high levels of unintended pregnancies, adolescent pregnancies, and high levels of maternal mortality indicate that access to FP services is a problem (4, 5, 18). The South African District Health Survey (SADHS) only identifies two aspects as access barriers – affordability and physical access (distance). The latest SADHS reports that only 2% of contraceptive users discontinued their contraceptive use because of a lack of access, specifically long distance; and 0.4% cited cost as the reason for discontinuation (18). These results provide a skewed

view of access barriers since the focus is only on availability and geographic access. Other sources have identified additional barriers, including poor QoC, limited variety of modern contraceptive methods, fragmented integration of sexual and reproductive health (SRH) services with PHC services, ailing infrastructure, shortage of trained nurses, poor attitudes of nurses, and stigma (4, 19-23). The SADHS results vary from other health services reports, such as the Household Survey (5). Hasumi and Jacobsen (24) reported that 43.8% of South Africans who relied on the public health care sector experienced problems in accessing care – which is concerning since they reported that over 80% of South Africans rely on the public health care sector for their health care needs. In addition, high levels of inequality to accessing health has been reported (25).

On a national and policy level, the SA government is dedicated to improving access to FP services and contraceptive methods by committing to the goals set out by FP2020 (26). The SA government has restructured contraceptive services provision, from a specialised vertical programme to a PHC integrated programme (27), and has implemented progressive FP policies (4, 28, 29), in order to address inadequacies in contraceptive access due to past history. However, how effective these policies are at improving access to contraception is unclear.

The integration of services - especially FP services with HIV care, is a key strategy proposed to improve access, inequity, and QoC (21, 30). This area has received much attention in recent years and there is a considerable focus to ensure that integration occurs (2, 20, 31, 32). However, for integration to be effective other barriers to accessing health care services (as mentioned above) must be addressed (WHO, 2008).

Based on these national survey results and trends, it is clear that accessing contraception methods and services is problematic – or at least access has not improved despite major policy reform and the reorganisation of SA health services to increase access. Despite the enabling policies and restructuring, the SA public health sector has struggled to make real gains in improving access to services, including FP service (24, 33, 34). Where these challenges lie within the larger concept of

access and which components are most significant remains unclear.

Methodology

Setting

Compared with other countries in the Sub-Saharan region, SA has an advanced total fertility rate (TFR, 2.6), relatively low unmet need for married women (14.9%), moderate modern contraceptive prevalence rate (mCPR, 47.9%), and high levels of general knowledge about available contraceptive methods amongst men and women (17, 18). However, numerous SRH challenges exist. Amongst these are high levels of unintended pregnancies (over half of all pregnancies are unintended), a high rate of unmet need amongst unmarried young women (27.9%), adolescent pregnancies (16% of women aged 15-19 years have had a pregnancy), high incidence of termination of pregnancies, and an alarming increase in the pregnancy-related mortality ratio from a 150 deaths per 100 000 live births in 1998 to 536 deaths per 100 000 live births in 2016 (4, 5, 18).

There is also a noticeable stagnation in contraceptive use and fertility trend rates in South Africa over the past two decades. South Africa's total fertility rate has declined from an estimated 6.0 in 1960 to 2.6 in 2016 (18, 35). But between 1998 and 2016 the TFR has remained almost stagnant. The same trend can be seen in the mCPR. Unmet need in married/in-union women has also stagnated at 15% in 1998 and 14.9% in 2016. However, unmet need for the sexually active group (regardless of marital status) has increased from 5.7% in 1998 to 18.7% in 2016 (18). Wanted and unintended pregnancy rates have also remained the same. In 1998 46% of births were wanted, 36% were mistimed, and 17% were not wanted - compared to 2016 where 46% were wanted, 34% were mistimed, and 20% were unwanted.

The study reported on in this paper was conducted in the eThekwini District of KwaZulu-Natal (KZN) province, SA. KZN is the second-most populous province in SA and is situated on the east coast of the country. The couple's year of protection rate (CYPR) is often used as a proxy indicator for the CPR.

The CYPR for KZN has consistently fallen below the national median, with the 2018/19 CYPR being 44.4% - a significant drop from the 2016/2017 CYPR of 66.1% (36). KZN also has the largest number of people living with HIV in the country, with over two million of the seven and a half million people living with HIV residing in the province (37). The eThekwini District is the third-largest metropolitan district in SA, with an estimated population size of three and a half million people. The CYPR for eThekwini is also below that of the national average at 44.4% for 2018/2019. Similar to the KZN province, eThekwini also has the largest number of people living with HIV per district in the country (37).

Study design

This study was conducted as part of formative work to inform the development of an intervention that aimed at increasing met need for modern contraception through community and HCP participation from a human rights perspective, in SA, Kenya and Zambia (the UPTAKE Project). Overall study results are presented by Cordero, Steyn (38). The detailed teamwork qualitative methodology is also published elsewhere (39). In this article, we report on findings from the South African data.

In-depth interviews (IDIs) and focus group discussions (FGDs) were conducted between 2015 and 2016, and purposive snowball sampling was used to recruit participants. In total, 127 participants participated from eThekwini District in KZN Province, SA.

Fourteen FGDs were conducted. Twelve of these FGDs were with community participants, and two with HCPs. Table 1 shows the breakdown of the FGD participants. IDIs were conducted with eight key informants (KIs) who were purposively selected based on their expertise in FP services, or community involvement. Four KIs were based within the communities from which the community members were recruited. The other four KIs held senior or programme level positions, specialising in the field of SRH and FP. Table 2 outlines the composition of the KIs.

Table 1: FGD participant breakdown

**FGDs conducted**	**No. of participants (n)**
1. Females, urban, teenagers (aged 15-19 years)	9
2. Females, rural, teenagers (aged 15-19 years)	10
3. Females, urban, young adults (aged 20-34 years)	8
4. Females, rural, young adults (aged 20-34 years)	10
5. Females, urban, adults (aged 35-49 years)	8
6. Females, rural, adults (aged 35-49 years)	7
7. Males, teenagers (aged 15-19 years)	10
8. Males, young adults (aged 20-34 years)	8
9. Males, adults (aged 35-49 years)	7
10. Females who are unmarried, single (20-34 years)	8
11. Females who are married/in a relationship >1-year (20-34 years)	10
12. Females with no children (who are not infertile) (18-49 years)	8
**Total community participants**	**103**
13. HCP from local health facilities (including management, professional nurses): Group 1	8
14. HCP from local health facilities (including enrolled nurses, counsellors, and other operational staff): Group 2	8
**Total HCP participants**	**16**

Table 2: Key Informant participant breakdown

**Key Informants**	**No. of participants (n)**
1. Education	1
2. Community Care Givers	2
3. Traditional Healer	1
4. Programme Managers working in sexual and reproductive health (SRHR)	4
**Total key stakeholders**	**8**

FGDs and IDIs were conducted by trained research team members who had experience with qualitative interviewing and who were fluent in the local languages (isiZulu and English). Interviewers were appropriately matched with gender, age, and professional position to facilitate discussions. Demographic data were collected from all participants and were descriptively analysed. FGDs were conducted at community-based facilities. Key informants were interviewed at locations convenient to them, ranging from research site offices to participants' homes.

Two data types were used to uncover information about accessing FP services and contraception - audio transcripts and drawings. Community participants were asked about their experiences in accessing family planning services and HCPs were asked about their experiences in providing family planning services and their perceptions about access via IDIs and FGDs. Both groups of participants were also asked to make simple drawings to capture their experiences in accessing and providing family planning services.

Audio recordings of the FGDs and IDIs were transcribed and translated from isiZulu to English where necessary. The transcripts were reviewed and checked for accuracy and any ambiguity in the translations were discussed and clarified to ensure accuracy of the translations. At the end of the project, the results were shared with community members, and there was a high degree of agreement with the results presented. This further contributed to the validity and accuracy of the data.

Interview guides contained key theme-specific questions that were tailored for each category or type of participant, including the female FGDs, male FGDs, HCP FGDs, and key informant IDIs. Similar key thematic questions explored understandings of family planning; knowledge, attitudes and practices; key barriers and enablers to family planning access; perceptions and definitions of QoC; and the role of community participation in family planning and contraceptive services.

Ethics approval and consent to participate

This study received World Health Organization (WHO) Ethics Review Committee (ERC) (Project ID A65896) and Research Project Review Panel (RP2) approval. Local country ethics review and approval were provided by the University of the Witwatersrand Human Research Ethics Committee (Health- HREC, reference number M1504101). Further ethics reciprocity was provided by the University of KwaZulu-Natal's Biomedical Research Ethics Committee (BREC). Permission was also obtained from the KwaZulu-Natal Provincial Department of Health to interview health care providers. All participants voluntarily signed an informed consent form, which included permission to audio record the interview/group sessions. Consent for minors (those aged <18years) was obtained from their identified parents or legal guardians. Assent was obtained from the minors themselves to participate.

Theoretical Frameworks

The AAAQ framework was adopted by FP2020 to measure access to FP services and contraception (13, 16). This framework is based on General Comment 14 and General Comment 22 on the Right to Health as drawn up by the United Nations Committee on Economic, Social, and Cultural Rights (3, 12). This framework consists of four components, two of which pertain to access and two of which relate more to QoC. The access components deal more with the obtainability of care, while the QoC components outline the way services should be delivered (16). The access elements include availability and accessibility; while the QoC aspects include acceptability and quality (3, 12, 16).

The AAAQ framework can be considered as a broader FP services use framework since it incorporates additional components that focus beyond just access to FP services. As argued previously, to gain a deeper understanding about access to FP services, a specific focus on access is required. For this reason, Penchansky and Thomas's (10) Access framework was used in this study to investigate access to FP services and contraceptive methods. They argue that specific dimensions must be identified in order to evaluate access effectively, and that those dimensions include availability, accessibility, affordability, accommodation, and acceptability (10). *Accessibility* refers to

the "relationship between location of supply and the location of clients"; *availability* refers to "the relationship of the volume and type of existing services (and resources) to the clients' volume and types of needs"; *accommodation* refers to the "relationship between the manner in which the supply resources are organized to accept clients […] and the clients' ability to accommodate to these factors and the clients' perception of their appropriateness"; *affordability* refers to the cost of services related to the clients' ability to pay; and *acceptability* which refers to the relationship of the attitude between clients' and providers (10 : 128). These five domains outlined by Penchansky and Thomas (1981) can be found in numerous other frameworks, including those that specifically focus on access to FP services. In this study, four of the components – availability, accessibility, affordability, and accommodation were found to address the access themes in the data. Acceptability, which refers to the attitudes between HCPs and clients, was found to relate to QoC and is not included in the results presented here, but will be presented elsewhere.

Social constructionism was used as the overall lens through which to view the data. Social constructionism, as a broad theory, postulates that humans construct concepts through which they can understand reality (40, 41). These constructs are often deeply embedded within respective cultures and shape the way humans understand and make sense of the world in which they live (41). Adopting a social constructionist stance towards the study of FP services and contraceptive use allows for a variety of voices to be heard. It is especially helpful when the opinions and knowledge of community members are sought as it allows for the expression of ideas and understanding by a variety of people.

Analysis

The audio transcripts and drawings underwent the same process of analysis. Firstly, a subset of the transcripts and drawings were read,reviewed and tested before a master codebook was developed by a cross-country team of researchers. During the initial round of coding, a subset of coded transcripts was double coded to increase the validity, reliability, and consistency of the findings (39).

Additional coding was done on the individual country level to elucidate themes specific to each country. NVivo (version 10, QSR International) was used as the computer-assisted qualitative data analysis software that facilitated coding and thematic analysis of the audio transcripts and drawings. A detailed account of the teamwork methodology is presented by Milford, Kriel (39).

Thematic content analysis was used to identify *a priori* and emergent themes. *A priori* themes were based on components from Penchansky and Thomas's (1981) Access framework described above. Inductive and emergent themes were further explored using the constant comparison method to allow for themes to emerge once data saturation was reached (42). Coding queries and matrix coding queries were run to explore the data further. Matrix coding queries were used to elucidate comparative themes between the various groups of participants and to show where coding queries intersected (43). These more complex queries allowed for in-depth exploration and to uncover larger themes embedded in the data.

Results

Demographic results – Community male and female participants

A total of 103 community members participated in this study. Seventy-eight of the community participants were female (75%), and 25 were male (25%). Reported adolescent pregnancies were high, with seven (37%) out of the nineteen adolescent females reporting pregnancy. Five out of the nine adolescent girls in the urban adolescent group reported pregnancy, compared to two out of ten in the rural adolescent group. No male adolescents reported a pregnancy with a female partner. Table 3 below shows the demographic details for the community participants.

Table 3: Demographic data for male and female community participants (N=103)

**Demographic Characteristics**	**Community males (n=25)**	**Community females (n=78)**
Age	23.8 years [15-40]	26.4 years [15-49]
**Educational levels**		
Incomplete secondary school level	84.0%, n=21	48.7%, n=38
Completed secondary school level	16.0%, n=4	51.3%, n=40
**Relationship status**		
Regular partner, >1yr, not living together	28.0%, n=7	62.0%, n=48
Regular partner, <1yr, not living together	28.0%, n=7	21.0%, n=16
Regular partner, >1yr, living together	8.0%, n=2	3.0%, n=2
Married	0	3.0%, n=2
Divorced	0	1.0%, n=1
Casual partner	16.0%, n=4	0
No current partner	4.0%, n=1	10.0%, n=8
Multiple partners	16.0%, n=4	0
**Pregnancy**		
Reported Pregnancy **(N=61)**	13.1%, n=8*	86.8%, n=53
Unplanned pregnancies	11.5%, n=7	73.7%, n=45
Planned pregnancies	1.6%, n=1	13.11%, n=8
Average age of first pregnancy [range]	22 years [20-24]	19.2 years [17-21]
None **(N=42)**	40.5%, n=17	59.5%, n=25
**Contraceptive method use (N=86, 83.5%)**		
Total reporting contraceptive use per sex	25.58%, n=22	74.41%, n=64
**Per method**$	N=22	N=64
Male condoms	77.27%, n=17	75%, n=48
Depot medroxyprogesterone acetate (DMPA) 3-monthly injection	27.7%, n=6*	29.7%, n=25
Norethisterone enanthate (NET-EN) 2-monthly injection	0*	7.8%, n=5
Pill	9.1%, n=2*	3.1%, n=2
Implant	4.5%, n=1*	10.9%, n=7
Emergency contraception	18.2%, n=4*	1.6%, n=1

**Reports female partner pregnancy/use*

$Values do not add to 100%.

Demographic results for health care providers and key stakeholders

HCP FGD group 1 (management and professional nurses) consisted of six female and two male participants. In HCP FGD group 2 (enrolled nurses, counsellors, and other operational staff), all the

participants were females (n=8). The Key Informants consisted of seven females and one male. All HCPs interviewed in this study worked at public primary health care clinics (PHC) or hospitals.

Table 4: Demographic data for the healthcare providers (N=16) and key informants (N=8).

**Demographic Characteristics**	**HCP group 1 (n=8)**	**HCP group 2 (n=8)**	**Key informants (n=8)**
Age	39.2 years [28-56]	37.6 years [26-47]	51.2 years [25-66]
Female	75%, n=6	100%, n=8	87.5%, n=7
Male	25%, n=2	0	12.5%, n=1
**Education**			
Incompleted secondary school level	0	0	25%, n=2
Completed secondary school level	0	0	12.5%, n=1
Tertiary incomplete	0	37.5%, n=3	0
Tertiary complete	100%, n=8	62.5%, n=5	62.5%, n=5
Average years of experience in current position	4.9 years	6.4 years	10 years

Thematic results

In this section, we explore the main themes that emerged from the data in relation to questions about access. These emergent themes were categorised per component of access, except for policy, which is a separate but crucial factor to access. Themes included: the implementation of policy; the current and preferred sources of FP services and modern contraception (accessibility); the availability of contraceptive methods and HCPs (availability); the ability to obtain modern contraceptive methods (affordability); and the organisation of services (including integration, appointment scheduling, and waiting times) (accommodation). Findings under the acceptability component related more to quality of care and will be reported elsewhere.

Implementation of policy

Participants described that the implementation of FP-related policy varied across service delivery points (SDPs). One female participant from the married/in-union group explained:

Clinics are not the same so policies are not the same because I once was in [name of place] … policies are not the same as ones from here. So it can happen that they allow young children to insert implant. That side [name of place] policies are tough ... So it might happen that it is their policies [at that place do] not to allow you [to use contraception] and say you are young, yet this side everything goes [and] children can do anything this side.

[Married Female FGD, 005]

<u>Accessibility</u>

Three sub-themes emerged from this theme – geographic access to current contraceptive sources, infrastructure, and preferred source of contraceptive methods.

- ***Current sources of FP services and contraceptive methods***

Community participants were asked to describe where they could obtain contraception in their community. To further explore this topic, participants were asked to make a simple drawing that reflected their experiences about where and how they access FP services and methods. Key elements that were asked to be included were their main source of obtaining contraception, the time travelled to the service, and their method of transport. They could also include any other aspects that they felt were relevant. Public PHCs were identified as the main source of modern contraceptive methods for people, as illustrated in the drawings (Table 5 and figures 1 to 5). Table 5 shows the data obtained from analysing the drawings per focus group.

Table 5 - Data from the community participants' drawings.

**Group**	**Mean age of group (years)**	**Modes of transport**	**Average travel time (minutes)**	**Sources of FP/C**
Female FGD urban adults (n=8)	41.8	Walking only (n=8)	Mean = 13<br>Range = [5;15]	Local PHC only
Female FGD urban young adults (n=8)	23.1	Walking only (n=7)<br>Walking and taxi (n=)	Mean = 29<br>Range = [10;60]	Local PHC only
Female FGD urban adolescents (n=9)	17.9	Walking only (n=7)<br>Public taxi only (n=1)<br>Walking and taxi (n=1)	Mean = 18<br>Range = [10;60]	Local PHC only
Female FGD rural adults (n=7)	38	Walking only (n=7)	Mean = 44<br>Range = [5; 60]	Local PHC only
Female FGD rural young adults (n=10)	25	Walking only (n=10)	Mean = 51<br>Range = [5; 120]	Local PHC only
Female FGD rural adolescents (n=10)	16.7	Walking only (n=10)	Mean = 66<br>Range = [30; 120]	Local PHC only
Female FGD women in union (n=10)	30.7	Walking only (n=6)<br>Walking and taxi (n=3)	Mean = 21<br>Range = [10; 30]	Local PHC only
Female FGD unmarried/single women (n=8)	26.6	Walking only (n=6)<br>Walking and taxi (n=1)	Mean = 45<br>Range = [13; 60]	Local PHC only
Female FGD women without children (n=8)	22	Walking only (n=7)<br>Public taxi only (n=1)	Mean = 26<br>Range = [10; 45]	Local PHC only
Male FGD adults (n=7)	37.6	Walking only (n=5)<br>Public taxi only (n=1)<br>Walking and taxi (n=1)	Mean = 31<br>Range = [5; 50]<br><br>Shop travel time<br>Mean = 9.2<br>Range [5;30]	Local PHC only (n=2)<br><br>Local PHC and shop (n=5)
Male FGD young adults (n=8)	21.3	Walking only (n=8)	Mean = 28<br>Range = [10; 45]<br><br>Shop travel time<br>Mean = 17.5<br>Range = [5;30]	Local PHC only (n=1)<br><br>Local PHC and shop (n=7)
Male FGD adolescents (n=10)	16.4	Walking only (n=10)	Mean = 86 minutes<br>Range = [5;240]	Local PHC only (n=9)<br><br>Shop only (n=1)

The main mode of transport reported across the groups was walking (n=92), followed by a mixed-use of transport of walking and using a taxi (n=7), and public taxi (n=4). Male participants included local shops rather than PHCs as a major source of FP methods, while female participants only drew their local PHCs. Below are examples of the drawings made by community participants. [Insert drawings, Figures 1 to 5 – or insert as an appendix?]

Insert Figure 1: Young adult male, P1

Insert Figure 2: Adolescent male, P2

Insert Figure 3: Rural Adult Female, P5

Insert Figure 4: Adult Female, no children, P1

Insert Figure 5: Married/In-union, adult female, P1

- ***Infrastructure***

HCP participants described the availability and accessibility of contraceptive methods at their healthcare facility, in relation to the available infrastructure. The physical size of the PHC determined if a separate room was available where FP services and care could be provided.

> *At my clinic there is a room specialising in family planning but all consultation rooms have family planning. They do it so that that there will not be long lines during the day because it [the family planning specific room] is closed at 16H00. After 16H00 service still continues and they can still find it in other [general care] rooms ...*
>
> *[HCP Group 1, 002]*

Smaller PHCs were constrained by the available space with the result that FP services were provided in all consulting rooms.

We do not have [a specific place if you want family planning] because our clinic is small. But all consultation rooms [have] all services…

[HCP Group 1, 003]

- ***Preferred source of FP services and contraceptive methods***

Participants were asked where they would prefer to obtain their contraception if they had a choice. While some participants were content with their local PHC, other sources were also identified. An alternative preferred source for accessing FP methods and services, often quoted, was the mobile clinic, as this female community participant explained:

[I]t could be better if there can be a mobile clinic because sometimes you don't have transport fare to go to the clinic because the clinic is far. Maybe the [Department of] Health can make means […] so that we can have easy access.

[Married Female FGD, P001]

Community participants also suggested that schools could be important sources from which to obtain modern contraceptive methods. One female adolescent participant noted her preference to obtain her contraceptive method from her school lavatory:

I would like to get them from school by the toilet (Group laughs), nobody knows what's happening inside there […]

[Rural Female Adolescent FGD, P010]

Others said that they would prefer to access FP/C from a private health care provider - especially from doctors. Young adult male participants expressed a preference to obtain condoms or SRH advise from male doctors:

F: Who would [you] like to get [contraception] from?

P003: From an experienced doctor who [is qualified for] these things.

F: Number 2.

P002: Doctor as well.

F: Number 1.

P001: I also say from the Doctor. Especially male one.

F: Male?

P001: Because a male […], he is going to understand […] how it works, because a female will [not] tell you that there are different condoms like this and that […]. He is going to tell you how [they] work because sizes are not the same. He is going to tell you that if you have this size this one works like this. So, it [is] better if it is a male doctor.

[Young Adult Male FGD]

Availability of contraceptive methods and health care providers

- ***Availability of contraceptive methods***

Both the community and HCP participants reported that short-acting and long-acting reversible contraceptive methods were readily available at local PHCs. However, access to a variety of contraceptive methods including longer-acting permanent methods (such as sterilisation) was described as being inaccessible, and therefore unavailable. One participant described the lengthy process involved in accessing female sterilisation:

Tubal ligation, [it is not available at the clinics] if you want to do it at the clinic it is a long process that I tried […] You first go to the clinic and get a referral letter so that you are able to go to [the hospital]. When you get to [the hospital] they start afresh and fill in forms and all that. It is a long process.

[Married Female Group, P008]

- ***Availability of trained HCPs***

Another sub-theme of availability was the limited availability of trained HCPs.

[I] can say that they [HCPs]… are not enough because it is not good that you walk a long distance to look for help and at the end you don't get it because […] there is little staff

[Community KI, P01]

One health KI raised the fact that HCP are not well trained on providing SRH care, including contraception:

The rights of the people are violated when it comes to family planning, sexual and reproductive health rights just by not providing nurses who are trained. It's a violation of people's rights.

[KI HCP, IDI, P7]

Affordability

In general, the affordability of contraceptive methods from PHCs was positively described as facilitating the uptake and use of contraception. Some minor reports about transport costs were raised by a few community participants – but this was not seen as a significant barrier. Two sub-themes emerged under affordability. The first was an individual user's ability to obtain services, and the second was the financial impact on male partners.

- ***Ability to access FP service and contraceptive methods.***

The user's ability to access FP services of their own choice – referred to as individual power by community participants, is the first sub-theme. Many community members discussed being constrained by their own financial situation to obtain contraception from a source of their own choice. One female participant explained:

F: How would you like to get these family planning and contraception? Where would you like to get them?

I don't have a choice - it is at the clinic because it is where I have power to reach but if I had

strength I would have loved to get from my doctor. […] But because of power you are forced to go to the clinic. But condoms you are able to buy if you have money [..] But like injection and what not you will not be able to buy.

[Urban Adult FGD, P006]

- ***Financial cost on male partners***

Male participants raised gendered perspectives about the economic expectations placed on them and the cost of accessing FP services and contraceptive methods. Some young adult male participants explained:

P4: It now costs her, she has to change the method she is using you see. It is like that my brother, when she changes it affects you financially, you see, as an "Outjie" (Guy) because she tells you "my brother […] it is you who is supposed to give her money", you see. To go and buy these things. Why, because they are not available at our local clinics.

P7: Yes it happens that she changes them.

[Young adult male FGD]

Adolescent males further described how gender conventions and relationship factors can influence the affordability of contraceptives.

P10: Ones that are available for free [referring to condoms] don't satisfy us.

I: They don't satisfy you?

P10: Because when you use it the lady complains that 'ayi, you look down on her because you are having sex with her using a [free government condom brand].[…] So you see that thing and then we are forced to buy [brand of condom] […]

P5: I also second number 10 he is really right, the minute you take out a [free government condom brand]on a girl, she gets the impression that you are not the guy that she thought you are you see. It means that you are forced to go and take [brand of condom], so that she can be satisfied that 'hah babe is having sex with me with such a thing' you see.

[Adolescent Male FGD]

Accommodation

Discussions about the organisation of FP services, which refers to the accommodative component of access, were raised in three-sub-themes: integrated care, accommodating missed visits, and waiting times.

- ***Integrated care***

Discussions surrounding the integration of the FP programme with PHC services raised numerous challenges with organising an efficient service that could accommodate a variety of client needs. This led to numerous community and HCP participants reporting that integration had a negative effect on accessing FP/C services.

HCP KIs who had been involved with family planning over a number of decades described how the integration with PHC services affected access to contraception in the current setting:

> *You know when family planning was introduced way back in the 1980's, '89… up to maybe the 2000. We had a service that we called state health services and at that time, family planning was working very well. But we've since now amalgamated all the primary health care services, that is where we actually miss the boat. [Because] in my opinion if we still maintained that family planning only service, maybe we wouldn't be having as many teenage pregnancies as we are having.*

[HCP KI, P05]

Community participants reported that they had good experiences with an NGO supported facility that focused only on FP services for young adults and adolescents, however this facility had been closed down. Participants – both community and HCP outlined the enabling environment that such a specific facility provided in terms of accessing FP services.

> *It was only family planning there at [name of facility], only those for family planning went there […]. It was fast, they closed it, […] And they were fast because you knew that you came*

for something that does not include anything else, it was a place for people on prevention only. Now they took us back to the clinic […]. That [is] the thing that made me [stop using] 2-monthly [injection] while [I was doing] well [on it].

[Females without children FGD, P003]

There was a lot of girls [at name of facility] they were doing family planning there. [It] was closed unfortunately, [and the clients] had to go to the clinic […] But we lost a lot of girls they stopped coming to be absorbed at the clinic because it was just for the youth only.

[HCP Group 1, P004]

- ***Accommodating missed visits***

The limited ability of PHCs to accommodate missed visits was raised by participants. Community participants pointed out that they often had compelling reasons for missing a scheduled date, mostly because of employment.

Sometimes there are compelling issues that make you miss your clinic date. […] then you find a temporary job [and] it is very hard to ask that you [can have the time off to] go to the clinic.

[Rural Adult Female FGD, P4]

Community participants reported that the consequences of missing an appointment date was often met with harsh treatment by the nurses:

[T]hings that they do the nurses if you have missed your date, they chase you away, while you were waiting that 3 hours and above. She will tell you to go and wait outside you will be the last to be attend[ed to], that is a turn off.

[Rural Adult Female FGD, P1]

- ***Waiting times***

Long waiting times was a much-discussed topic that participants related to the QoC, the accommodative ability of a PHC, and as a factor that contributed to their economic burden to obtain

contraception. Community participants described having to wait for a number of hours and that long waiting times could result in the discontinuation of contraceptive use.

Yes, I also left it [referring to method discontinuation] because of that, the 2-months [injection], it was treating me well […]The thing that made me stop the 2-months [injection] is that thing of the nurses, they sometimes attend to you and [sometimes] they do not attend to you […] You sit there the whole day at the clinic. You will arrive in the morning at 10, 11 you will leave at 4 not [being] attended to, it's just busy. What's worse is they mix you with the sick [clients] sometimes while they said, there is a place that used to be called 'be injected' [the specialised NGO supported FP service that was closed].

[Female Group without Children, P3]

One female participant explained that even though there may only be a few clients seeking FP services, they still have to wait for lengthy times:

Because we sit more than 3 hours at the clinics when you're there to take injection. Sometimes you find that you are 4 [clients] who are there for contraceptives.

[Rural Adult Female FGD, P1]

HCPs gave reasons for the long waiting times, but also felt that they impacted on quality of care:

[O]ur clients they wait for a long time to be seen because they need to be taught about the family planning then from there, they come back to the queue. So, it's not good quality for the clients […].

[HCP Group 1, FGD, P5]

Another problematic factor raised about time, was that in some PHCs adolescent school children were attended to before adult clients, which resulted in tensions, and services not being accessed:

They usually start with children, yet you are also in a hurry to get to your own work as a domestic worker in the suburbs […]. You end up taking your card, put it in your bag and leave.

[Rural Adult Female FGD, P4]

Discussion

In this paper, access to FP services and modern contraceptive methods were explored from a community and health care provider perspective. Penchansky and Thomas's (1981) definition and components of access were employed in this qualitative study to identify the key dimensions of access. Inductive emergent sub-themes were categorised according to the components of access. Under the accessibility theme, geographic accessibility, infrastructure, and preferred sources were discussed. The availability theme consisted of the availability of contraceptive methods and trained providers. The affordability theme was in general positively described but included two important sub-themes – the ability to obtain contraception and the gendered expectation that male partners should provide financial support to access contraceptive methods. The last theme was accommodation, which included the integration of services, accommodating missed visits, and waiting times as sub-themes. Acceptability which is a component of the access framework refers to the attitudes and interpersonal relationship between providers and clients. This definition was more in line with QoC and was therefore not discussed in this paper, but will be addressed elsewhere.

Policy

Access to health care services begins with policy, so although it is not a component of access, it is essential to the concept of access (10, 44). SA has progressive SRH policies that place human rights at the centre (4). However, effective policy depends on the consistent and accurate implementation of those policies (20). The findings from this data showed that FP policies are not consistently implemented across all PHCs, resulting in decreased access for some potential contraceptive users. The reason for the varied implementation could be due to HCPs not being appropriately consulted prior to policy updates, or on strategies suggested to implement changes (20). Buy-in from frontline HCP is essential to expand policy-based access to FP services and contraceptive methods (19).

The varied application of policies could also be as a result of different levels of services being offered by different facilities and HCPs, according to policy regulations. For instance, sterilisation procedures

are only available at hospitals and not PHCs, and the Implant can only be inserted by a trained nurse or doctor (45). If a smaller facility does not have such a specialist-trained HCP, then the availability of those methods and services are limited. Misinterpretation about service availability could also result in community members assuming that HCPs are applying policies on an ad hoc basis. This points to a need for greater engagement between community members and HCPs.

Accessibility of FP services and methods

The first component of the Access framework highlighted in this data, is the accessibility component that is defined as: "the "relationship between location of supply and the location of clients" (10: 128). This component was discussed through three sub-themes: geographic accessibility, infrastructure, and preferred sources of contraception.

Overall, the accessibility of FP services and contraceptive methods was positively associated with access. Participants echoed the reported findings from previous research, (5, 18) that PHCs were the main source of contraception. Participants' drawings showed that access is easier for some community members than others. Adolescent male participants reported the longest travelling time, while adult male participants reported the shortest travelling time by identifying local shops as an alternative source of contraceptive method (male condoms). Female participants identified PHCs as the only source to access contraception. This interesting gender dynamic raises issues of equity as it is easier to obtain male condoms from local shops, where the government-branded condoms are freely available, than to obtain other forms of contraception from the PHCs. Unless females obtain these free male condoms from local shops, they have no choice but to engage with their local PHC to obtain contraception. It was unclear why women did not recognise local shops, or other facilities such as pharmacies, as possible sources for contraception and requires further exploration.

HCPs discussed how their infrastructure impacted on accessing FP services. Some HCPs reported that

their PHC facilities do not have sufficient space or adequate infrastructure to provide separate FP services, thereby constraining access to contraception for some FP clients. Ailing infrastructure has also been shown to have a negative impact on the psychological well-being of HCPs, which further limits access to services (20, 46).

The choice of provider also plays a significant role in the satisfaction, uptake, and continued access to FP services. Penchansky and Thomas (10) showed in their work that having the same HCP of choice is an important factor for retention in care and satisfaction with availability. This preference was demonstrated in this study, whereby community members expressed their desire to access contraception from an HCP of their own choice, especially for male participants who voiced their preference for a male HCP.

Availability of contraceptive methods and trained HCPs

The second access theme is availability, which is defined as “the relationship of the volume and type of existing services (and resources) to the clients’ volume and types of needs” (10: 128). While participants reported that modern contraceptive methods were available, these were usually limited to the shorter-acting methods. Of importance was the dissatisfied reports by female participants about the unattainability of longer acting and permanent methods such as sterilisation.

The biggest concern noted in this data in relation to the availability component was the shortage of trained HCPs. This in itself is not a new finding (47, 48), but it highlights the need for human resources to be included in discussions about the availability of contraception. Currently in SA, only qualified registered nurses and medical doctors can provide hormonal or long-acting contraceptive methods such as the IUD (45). The data presented here showed the effect that staff shortages have on the waiting times and user satisfaction with FP service access. In addition, due to the restructuring of the general health care sector from a hospital-based to primary health care orientated services, nursing training has shifted from specialised care to more generalist care (46).

As a result, nurses who specialised in FP care have fallen away, thereby reducing the number of nurses who can provide contraception. The introduction of the Implanon NXT Implant has highlighted the importance of having adequately trained nurses to expand access to a variety of contraceptive methods, as inadequate training resulted in botched removals of the Implant, resulting in decreased uptake of this contraceptive method (19).

Affordability of accessing FP services and contraceptive methods.

The affordability component refers to the cost of services as related to a client's ability to pay and includes the direct and indirect costs of obtaining contraception (10). FP services and contraceptive methods are provided free-of-charge at public sector PHCs, and this was reported as a positive factor in the uptake of contraception. The aspect of affordability that had the biggest effect on accessing contraception reported by participants, was the individual's economic power to access services from a provider of their choice. This finding ties in with availability. As reported above, the individual ability to choose a provider is crucial for the satisfaction and continued use of health care services (49), and particularly relevant for contraceptive use as it is a long-term health strategy. This finding presents an almost insurmountable challenge for the district-based PHC model through which FP services are provided in the public health care sector of SA. As explained by participants in this study, in this type of system, women have no choice but to access their contraception from their nearest PHC. They have limited autonomy about which HCP they would like to receive care from, unless they have the economic means to obtain contraception from a private-paid HCP.

Indirect costs were also mentioned in this study. Male participants discussed the economic burden placed on them to provide their female partners with money to obtain contraception. Another financial concern was raised by community members who were employed and found the PHC operating hours to conflict with their working schedules. Women reported that because of their employment they were willing to discontinue contraceptive method use due to the inability of PHCs to accommodate them.

Accommodation – the organisation of current FP services in PHC

The first three components of access – accessibility, availability, and affordability are the most commonly associated components of access (50). The Penchansky and Thomas (1981) access framework also includes accommodation and acceptability as part of access. Accommodation is defined as "the relationship between the manner in which the supply resources are organized to accept clients (including appointment systems, hours of operation, walk-in facilities, telephone services) and the clients' ability to accommodate these factors and the clients' perception of their appropriateness"; while acceptability refers to the relationship of the attitude between clients' and providers (10: 128). The acceptability component is more appropriately addressed under discussions related to quality of care and will be reported on in a subsequent article.

Accommodation and the manner in which FP services are organised was identified as the most significant component of accessing FP services and contraception in this setting. Three sub-themes emerged– integration, accommodating missed visits, and long waiting times.

The integration of FP services with PHC and particularly HIV care is part of the SGD target 3.7 and is included as a goal in the SA family planning policy (1, 45). Restructuring the specialised vertical FP programme to integrate with PHC was one of the most significant changes to the delivery of modern contraception in SA (20, 21, 27, 51) Despite almost two decades of attempting to integrate FP services with district-based PHC services, full implementation is still lagging, yet the importance and need for successful integration are clear (20, 32, 52, 53).

A shared view held by community and HCP participants, in this study, was that the integration of FP services with PHCs hampered access to contraception and decreased their overall satisfaction with care. There was an overall preference for a specialised FP service that would only provide contraception. The most significant reason cited for the dissatisfaction with integrated services was the resulting over-crowded PHC facilities that contributed to lengthy waiting times. Neither of these key concerns are currently addressed by any model of integration. Long waiting times is one of the

most commonly reported problems related to accessing PHC services, including FP services, in SA, and was reflected in this data (24, 54). McIntyre and Chow (55) found in their review that waiting times are often misreported and highlight the need for further exploration in the context of primary health care settings. They also found that long waiting times present as a significant barrier to accessing care (55).

Scheduling appointments is a clear strategy to improve the ability of a PHC to accommodate clients. A challenge arises when clients do not return to the PHC on their follow-up date. Community participants reported that they occasionally miss their appointment dates, often due to work requirements. This highlights the restrictive accommodating capacity of operating hours of a PHC, which is usually from 08:00 am to 16:00 pm. In addition, it has been found that in some South African PHCs, nurses only attend to clients in the mornings, reserving afternoons for administrative work, thereby further limiting operating hours (21).

Missed visits for contraceptive continuation appointments are common in this setting. Baumgartner et al (56) reported that nearly 50% of DMPA users return late for their follow-up appointment, with some being denied access to follow-up DMPA injections. One of the motivations to introduce LARCS such as the Implanon NXT Implant is to address the follow-up issue and relieve some of the over-crowding pressure on PHCs (4, 19). However, since DMPA remains the most popular hormonal contraceptive in SA, PHCs must consider innovative strategies to accommodate a variety of client needs, especially for those FP users who are employed.

In this data, long waiting times were reported as a key reason for discontinuing contraception – one of the few directly related to accessing FP services – and the most significant sub-theme reported in this data. Participants reported that numerous factors contributed to lengthy waiting times, including the integration of FP services with PHC general services and the shortage of HCPs. The National Department of Health suggest that the average waiting time in a PHC should be two hours (57) – but participants in this study reported waiting four hours or more just to receive

contraception. Addressing long waiting times in PHCs is a complex problem that will require adjustments in other important areas such as having sufficient HCPs, ensuring that capacity is not over-extended, and adjusting the operating hours of PHCs.

Limitations and strengths

Some limitations exist for this study. Firstly, the data presented here are based on experiences with the public health care sector only. For a more comprehensive comparison of access to contraception data and experiences from private health care users should also be evaluated. Secondly, as this was a qualitative study, the results are not necessarily generalisable. Instead, the focus is rather on the transferability of the method and findings to other settings. While the findings are specific to the South African context, and specifically KZN, the method for evaluating access using a holistic approach and identifying the relevant components is relevant for other settings.

Using a community perspective is a key contribution that this paper makes to the literature. Usually, the perspective of facility clients is used to evaluate the satisfaction with services, which can be skewed toward positive overreporting out of fear of repercussions (58). Interviewing community members who may or may not be FP clients provide an opportunity for a less-influenced perspective and evaluation on accessing FP services.

A number of questions about accessing FP services remained unanswered in this data set and require further exploration. Adolescent males reportedly had the longest travel time to FP services, but it was not well explained in the data as to why. Female participants did not identify any other sources for contraception other than PHCs – this needs exploration. The long waiting times were a significant issue and require urgent further exploration in expanding integration of care.

Conclusion

This paper sheds light on how community members and (HCPs) evaluate access to FP services and contraceptive methods. In contrast to the results presented by the SADHS (2016), the data showed that significant barriers to accessing contraception exist in this setting which can result in contraceptive discontinuation. Overall, the participants were satisfied with the accessibility and affordability components. They were less satisfied with the availability of a range of contraceptive methods and the limited number of trained providers were raised as a concern. Accommodation was identified as the component that presented the most significant barriers to accessing FP services. In particular, the ineffective organisation of services resulted in lengthy waiting times that could result in the discontinuation of FP use. The addition of the male perspective in accessing contraception was a new finding on this subject. Men not only described the financial burden of accessing FP services but also highlighted the importance of having alternative sources of contraception in the communities that are easily accessible.

The importance of recognising that access is a multidimensional concept and that the most important components should be identified in order to gain a clear understanding about the challenges in accessing contraception was made clear. Careful attention must also be paid to policy implementation over time, as local contextual changes could make policies redundant. The effectiveness of policies should be evaluated as was made clear in the discussions about integration of care. Women face many obstacles when accessing (5) contraception, the argument here is that the health care system itself should not be an obstacle. Every effort should be made to ensure that all women who need or want contraception should be able to get it without much hassle or having to overcome numerous barriers.

Even though the data for this study were collected prior to the onset of the COVID-19 pandemic the findings highlight the importance of paying attention to all the components of access. Health care facilities and systems must identify where access problems exist and address them appropriately in

order to be prepared for unexpected new epidemics that require the full functioning of health care services.

Statements:

Data availability: The database is not publicly available as it contains information that could compromise research participant privacy/consent. However, some anonymised aspects of the datasets may be available upon request and with permission of the Department of Reproductive Health and Research, World Health Organisation. Note that data sharing is subject to WHO data sharing policies and data use agreements with the participating research centres.

Authorship:

Study Conceptualization: Petrus S. Steyn, Jennifer Ann Smit, Joanna Paula Cordero

Data curation: Yolandie Kriel, Cecilia Milford, Joanna Paula Cordero

Formal analysis: Yolandie Kriel, Cecilia Milford

Funding acquisition: Petrus S. Steyn, Joanna Paula Cordero, Jennifer Ann Smit,

Investigation: Yolandie Kriel, Cecilia Milford, Joanna Paula Cordero, Petrus S. Steyn, Jennifer Ann Smit

Methodology: Petrus S. Steyn, Joanna Paula Cordero, Jennifer Ann Smit

Validation: Yolandie Kriel, Cecilia Milford

Writing original draft: Yolandie Kriel

Reviewing & editing: Yolandie Kriel, Cecilia Milford, Joanna Paula Cordero, Petrus S. Steyn, Jennifer Ann Smit, Fatima Suleman

Financial disclosure: This work received funding through a grant received by UNDP-UNFPA-UNICEF-

WHO-World Bank Special Programme of Research, Development and Research Training in Human Reproduction (HRP), a cosponsored programme executed by the World Health Organization (WHO) from the Bill and Melinda Gates Foundation [OPP1084560] and the United States Agency for International Development (USAID) through the USAID/WHO Umbrella Grant 2016-2018.

Ethics statement: This study received World Health Organization (WHO) Ethics Review Committee (ERC) (Project ID A65896) and Research Project Review Panel (RP2) approval. Local country ethics review and approval was provided by the University of the Witwatersrand Human Research Ethics Committee (Health- HREC, reference no 1504101). Further ethics reciprocity was provided by the University of KwaZulu-Natal's Biomedical Research Ethics Committee (BREC). Permission was also obtained from the KwaZulu-Natal Provincial Department of Health to interview health care providers. All participants voluntarily signed an informed consent form, which included permission to audio record the interview/group sessions. Consent for minors (those aged <18years) was obtained by their identified parents or legal guardians. Assent was obtained from the minors themselves to participate.

Disclaimer: The authors alone are responsible for the views expressed in this article and they do not necessarily represent the views, decisions or policies of the institutions with which they are affiliated.

References

1. UN. The Sustainable Developmental Goals 2015 [Available from: https://sustainabledevelopment.un.org/?menu=1300.

2. FP2020. FP2020 - Women at the center 2018-2019. Family Planning 2020; 2019.

3. General Comment No. 22 on the right to sexual and reproductive health (artilce 12. of the International Covenant on Economic, Social and Cultural Rights), (2016).

4. Lince-Deroche N, Pleaner M, Harries J, Morroni C, Mullick S, Firnhaber C, et al. Achieving universal access to sexual and reproductive health services: the potential and pitfalls for contraceptive services in South Africa. Durban: Health Systems Trust; 2016. Report No.: 1025-1715.

5. Chersich M, Wabiri N, Risher K, Shisana O, Celentano D, Rehle T, et al. Contraception coverage and methods used among women in South Africa: A national household survey. South African Medical Journal. 2017;4(107):pp. 307-14.

6. FP2020. FP2020 Data Dashboard 2020 [Available from: http://www.familyplanning2020.org/data-dashboard.

7. Bongaarts J, Casterline J. Fertility Transition: Is sub-Saharan Africa Different? Population and development review. 2013;38(Suppl 1):153-68.

8. Singh S, Darroch JE, Ashford LS. Adding it up: The costs and benefits of investing in sexual and reporductive health 2014 New York: Guttmacher Institute United Nations Population Fund (UNFPA); 2014 [Available from: http://www.unfpa.org/sites/default/files/pub-pdf/Adding%20It%20Up-Final-11.18.14.pdf.

9. UNDESA. World Fertility and Family Planning 2020. Population Division of the United Nations Department of Economic and Social Affairs; 2020.

10. Penchansky R, Thomas JW. The Concept of Access: Definition and Relationship to Consumer Satisfaction. Medical Care. 1981;19(2):127-40.

11. Hardee K, Kumar J, Newman K, Bakamjian L, Harris S, Rodríguez M, et al. Voluntary, Human Rights–Based Family Planning: A Conceptual Framework. Studies in Family Planning. 2014;45(1):1-18.

12. UNCESCR. United Nations Committee on Economic Social and Cultural Rights. General Comment No. 14, The right to the highest attainable standard of health (Article. 12 of the International Covenant on Economic, Social and Cultural Rights). Report on the Twenty-Second, Twenty-Third, and Twenty-Fourth Sessions, Economic and Social Council Official Records, 2001, Supplement No. 2. 2000.

13. Choi Y, Fabic MS, Adetunji J. Measuring Access to Family Planning: Conceptual Frameworks and DHS Data. Studies in Family Planning. 2016;47(2):145-61.

14. Bertrand JT, Hardee K, Magnani RJ, Angle MA. Access, quality of care and medical barriers in family planning programs. International family planning perspectives. 1995:64-74.

15. Stephenson R, Beke A, Tshibangu D. Community and Health Facility Influences on Contraceptive Method Choice in the Eastern Cape, South Africa. International Family Planning Perspectives. 2008;34(2):62-70.

16. Jain AK, Hardee K. Revising the FP Quality of Care Framework in the Context of Rights-based Family Planning. Studies in Family Planning. 2018;0(0).

17. Caldwell JC, Caldwell P. Africa: The New Family Planning Frontier. Studies in Family Planning. 2002;33(1):76-86.

18. NDoH, StatsSA, SAMRC, ICF. South Africa Demographic and Health Survey 2016 - Full report. Pretoria South Africa; Rockville Maryland: National Department of Health, Statistics South Africa, South African Medical Research Council, ICF; 2019.

19. Adeagbo OA, Mullick S, Pillay D, Chersich MF, Morroni C, Naidoo N, et al. Uptake and early removals of Implanon NXT in South Africa: Perceptions and attitudes of healthcare workers2017.

20. McIntyre D, Klugman B. The Human Face of Decentralisation and Integration of Health Services: Experience from South Africa AU - McIntyre, Di. Reproductive health matters. 2003;11(21):108-19.

21. Milford C, Scorgie F, Rambally Greener L, Mabude Z, Beksinska M, Harrison A, et al. Developing a model for integrating sexual and reproductive health services with HIV prevention and care in KwaZulu-Natal, South Africa. Reproductive Health. 2018;15(1):189.

22. Pleaner M, Morroni C, Smit J, Lince-Deroche N, Chersich MF, Mullick S, et al. Lessons learnt from the introduction of the contraceptive implant in South Africa2017.

23. Harries J, Constant D, Wright V, Morroni C, Müller A, Colvin CJ. A multidimensional approach to inform family planning needs, preferences and behaviours amongst women in South Africa through body mapping. Reprod Health. 2019;16(1):159.

24. Hasumi T, Jacobsen KH. Healthcare service problems reported in a national survey of South Africans. International Journal for Quality in Health Care. 2014;26(4):482-9.

25. Harris M, Harris E, Roland M. Access to primary health care: three challenges to equity. Aust J Prim Health. 2004;10:21 - 9.

26. FP2020. Family Planning 2020 commitment - South Africa. London Summit on Family Planning 20122012.

27. Van Rensburg H, Engelbrecht M. Transformation of the South African health system: post-1994. In: Van Rensburg H, editor. Heatlh and Health Care in South Africa. second ed. Pretoria: Van Schaik; 2012. p. 121 - 82.

28. Ndinda C, Ndhlovu T, Khalema NE. Conceptions of Contraceptive Use in Rural KwaZulu-Natal, South Africa: Lessons for Programming. International Journal of Environmental Research and Public Health. 2017;14(4):353.

29. Ndegwa DG. International thinking on population policies and programmes from Rome to Cairo: Has South Africa kept pace? Southern African Journal of Demography. 1996;6(1):49-56.

30. WHO. Quality of care: a process for making strategic choices in health systems. Geneva: World Health Organization; 2006.

31. Cooper D, Mantell JE, Moodley J, Mall S. The HIV epidemic and sexual and reproductive health policy integration: views of South African policymakers. BMC Public Health. 2015;15:217.

32. Uebel K, Guise A, Georgeu D, Colvin C, Lewin S. Integrating HIV care into nurse-led primary health care services in South Africa: a synthesis of three linked qualitative studies. BMC Health Services Research. 2013;13(1):1-11.

33. Ataguba JE-O, McIntyre D. Who benefits from health services in South Africa? Health Economics, Policy and Law. 2013;8(1):21-46.

34. Harris B, Goudge J, Ataguba JE, McIntyre D, Nxumalo N, Jikwana S, et al. Inequities in access to health care in South Africa. Journal of Public Health Policy. 2011;32:S102-S23.

35. Moultrie TA, Timaeus IM. The South African Fertility Decline: Evidence from Two Censuses and a Demographic and Health Survey. Population Studies. 2003;57(3):265-83.

36. Massyn N, Barron P, Day C, Ndlovu N, Padarath A. District Health Barometer 2018/19. Durban: Health Systems Trust; 2020 February 2020.

37. UNAIDS. AIDSinfo - South Africa: UNAIDS; 2020 [Available from: www.aidsinfo.unaids.org

38. Cordero JP, Steyn PS, Gichangi P, Kriel Y, Milford C, Munakampe M, et al. Community and provider perspectives on addressing unmet need for contraceptives. 2017.

39. Milford C, Kriel Y, Njau I, Nkole T, Gichangi P, Cordero JP, et al. Teamwork in Qualitative Research:Descriptions of a Multicountry Team Approach. International Journal of Qualitative Methods. 2017;16(1):1609406917727189.

40. Schwandt TA. Three Epistemological Stances for Qualitative Inquiry: Interpretivism, Hermeneutics, and Social Constructionism. In: Denzin N, Lincoln YS, editors. The Handbook of Qualitative Research. Second ed. Thousand Oak, California, London, New Delhi: Sage Publications; 2000. p. 189-213.

41. Burr V. Social constructionism. East Sussex, New York: Routledge; 2015.

42. Ryan GW, Bernard HR. Techniques to Identify Themes. Field Methods. 2003;15(1):85-109.

43. Bazeley P, Jackson K. Qualitative data analysis with NVivo. Los Angeles [i.e. Thousand Oaks, Calif.]; London: SAGE Pubications; 2013.

44. Aday LA, Andersen R. A framework for the study of access to medical care. Health services research. 1974;9(3):208-20.

45. NDoH. South African National Contraception and Fertility Planning Policy and Service Delivery Guidelines: A companion to the National Contraception Clinical Guidelines. In: Department of Health, editor. Pretoria: National Department of Health; 2012.

46. van Rensburg H, Heunis J, Steyn F. Human resources for health and the health professions in South Africa. In: HCJ vR, editor. Health and Health Care in South Africa. Second edition ed. Pretoria: Van Schaik Publishers; 2012. p. 361-424.

47. Mayosi BM, Lawn JE, van Niekerk A, Bradshaw D, Abdool Karim SS, Coovadia HM. Health in South Africa: changes and challenges since 2009. The Lancet. 2012;380(9858):2029-43.

48. Mayosi BM, Benatar SR. Health and health care in South Africa—20 years after Mandela. New England Journal of Medicine. 2014;371(14):1344-53.

49. Thomas JW, Penchansky R. Relating Satisfaction with Access to Utilization of Services. Medical Care. 1984;22(6):553-68.

50. Gulliford M, Figueroa-Munoz J, Morgan M, Hughes D, Gibson B, Beech R, et al. What does 'access to health care' mean? Journal of Health Services Research & Policy. 2002;7(3):186-8.

51. Kaufman CE. Reproductive Control in Apartheid South Africa. Population Studies. 2000;54(1):105-14.

52. Maharaj P, Cleland J. Integration of sexual and reproductive health services in KwaZulu-Natal, South Africa. Health Policy and Planning. 2005;20(5):310-8.

53. Adeniyi OV, Ajayi AI, Moyaki MG, Goon DT, Avramovic G, Lambert J. High rate of unplanned pregnancy in the context of integrated family planning and HIV care services in South Africa. BMC Health Services Research. 2018;18:1-.

54. Jacobsen KH, Hasumi T. Satisfaction with healthcare services in South Africa: results of the national 2010 General Household Survey. The Pan African medical journal. 2014;18:172.

55. McIntyre D, Chow CK. Waiting Time as an Indicator for Health Services Under Strain: A Narrative Review. INQUIRY: The Journal of Health Care Organization, Provision, and Financing. 2020;57:0046958020910305.

56. Baumgartner JN, Morroni C, Mlobeli RD, Otterness C, Myer L, Janowitz B, et al. Timelines of contraceptive reinjections in South Africa and its relation to unintentional discontinuation. International Perspectives on Sexual & Reproductive Health. 2007;33(2):66-74.

57. National Policy on Management of Patient Waiting Time in Out Patient Departments, (2015).

58. Tumlinson K, Speizer IS, Davis JT, Fotso JC, Kuria P, Archer LH. Partner Communication, Discordant Fertility Goals, and Contraceptive Use in Urban Kenya. African Journal of Reproductive Health / La Revue Africaine de la Santé Reproductive. 2013;17(3):79-90.

Figure

Click here to access/download;Figure;Y Kriel Paper 3 figures PLOS ONE 31May21.docx

Y Kriel Figures

Figure 1: Young adult male, P1

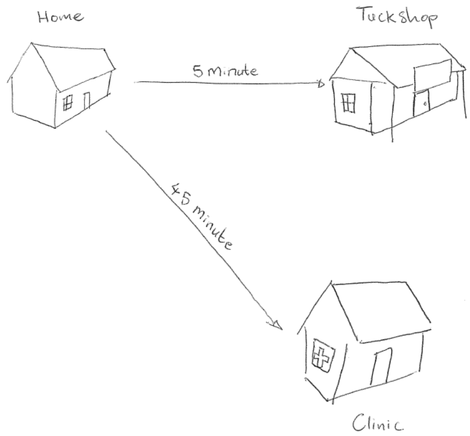

Figure 2: Adolescent male, P2

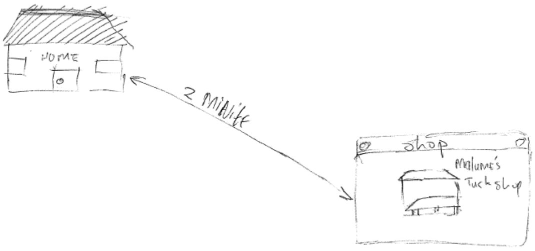

Figure 3: Rural Adult Female, P5

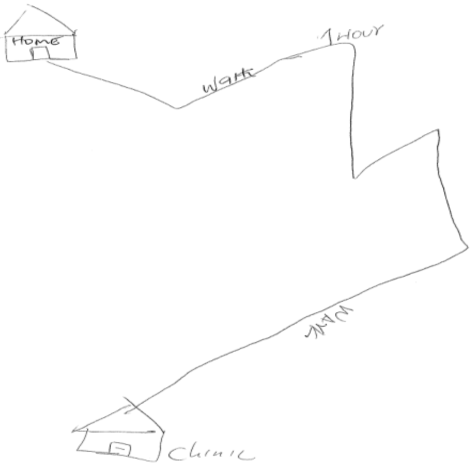

Figure 4: Adult Female, no children, P1

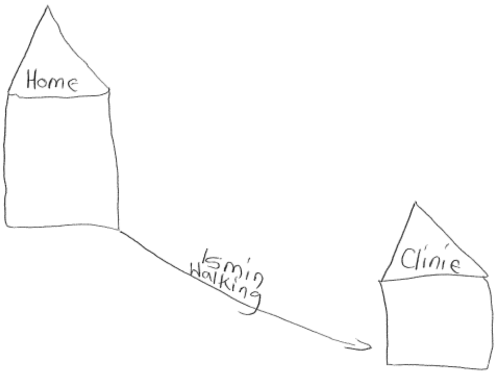

Figure 5: Married/In-union, adult female, P1

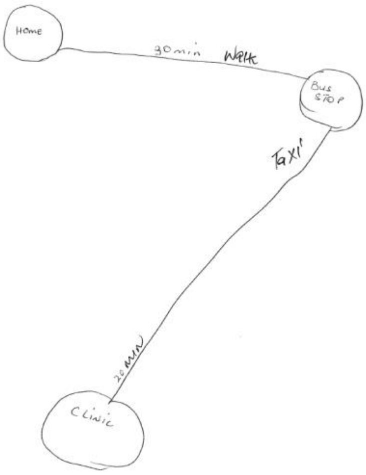

Related published article

Click here to access/download
Supporting Information
Kriel_et_al-2019-Reproductive_Health.pdf
