# Supplementary material for: Access to public sector family planning services and modern contraceptive methods in South Africa: A qualitative evaluation from community and health care provider perspectives
Source: PLoS One. 2023 Mar 17;18(3):e0282996. doi: 10.1371/journal.pone.0282996 (PMC10022780; doi:10.1371/journal.pone.0282996)
Supplement: Attachment — Submitted filename: Responses to Reviewers.docx [file pone.0282996.s009.docx]

**Response to reviewers**

Thank you to the reviewers and the editor for your comments. We have addressed all the issues that were raised and responded to your suggestions as we deemed appropriate.

When submitting your revision, we need you to address these additional requirements.

1. **Please ensure that your manuscript meets PLOS ONE's style requirements, including those for file naming. The PLOS ONE style templates can be found at https://journals.plos.org/plosone/s/file? id=wjVg/PLOSOne_formatting_sample_main_body.pdf and https://journals.plos.org/plosone/s/file? id=ba62/PLOSOne_formatting_sample_title_authors_affiliations.pdf**

- **Response:**

Thank you, the formatting was updated as per the journal style.

2. **Please include a copy of the interview guide used in the study, in both the original language and English, as Supporting Information, or include a citation if it has been published previously.**

- **Response:**

The interview guides are included now as part of the supporting information.

3. **We note that the grant information you provided in the ‘Funding Information’ and ‘Financial Disclosure’ sections do not match. When you resubmit, please ensure that you provide the correct grant numbers for the awards you received for your study in the ‘Funding Information’ section.**

- **Response:**

The funding information will be updated.

4. **We noted in your submission details that a portion of your manuscript may have been presented or published elsewhere. Table 1 ,2 , 3 and 4 Please clarify whether this publication was peer-reviewed and formally published. If this work was previously peer- reviewed and published, in the cover letter please provide the reason that this work does not constitute dual publication and should be included in the current manuscript.**

- **Response:**

The tables were removed to reduce any dual publication. Careful attention was paid to avoid any dual publication among the manuscripts that arise from this dataset. Some information like the demographic data might be similar due to the nature of such data. All other text and findings presented are unique to each paper.

As explained in the original cover letter and submission this paper forms part of a larger project, The UPTAKE Project, and the main author’s PhD thesis.

5. **We note that you have indicated that data from this study are available upon request. PLOS only allows data to be available upon request if there are legal or ethical restrictions on sharing data publicly. For more information on unacceptable data access restrictions, please see** [**http://journals.plos.org/plosone/s/data-availability#loc-unacceptable-data-**](http://journals.plos.org/plosone/s/data-availability#loc-unacceptable-data-) **access-restrictions.**

**In your revised cover letter, please address the following prompts:**

a) **If there are ethical or legal restrictions on sharing a de-identified data set, please explain them in detail (e.g., data contain potentially sensitive information, data are owned by a third-party organization, etc.) and who has imposed them (e.g., an ethics committee). Please also provide contact information for a data access committee, ethics committee, or other institutional body to which data requests may be sent.**

- **Response**

The database is not publicly available as it contains information that could compromise research participant privacy and consent. However, some anonymised aspects of the datasets may be available upon request and with the permission of the Department of Sexual and Reproductive Health and Research, World Health Organization, and the MatCH Research Unit (MRU). Note that data sharing is subject to WHO data sharing policies and data use agreements with the participating research centre, MRU. For permission to access the database, please contact the MatCH Research Unit at [info@mru.ac.za](mailto:info@mru.ac.za)

b) **If there are no restrictions, please upload the minimal anonymized data set necessary to replicate your study findings as either Supporting Information files or to a stable, public repository and provide us with the relevant URLs, DOIs, or accession numbers. For a list of acceptable repositories, please see** [**http://journals.plos.org/plosone/s/data-**](http://journals.plos.org/plosone/s/data-) **availability#loc-recommended-repositories.**

**We will update your Data Availability statement on your behalf to reflect the information you provide.**

6. **Your ethics statement should only appear in the Methods section of your manuscript. If your ethics statement is written in any section besides the Methods, please delete it from any other section.**

- **Response:**

Thank you, the ethics statement was removed from the statements section.

7. **We note you have included a table to which you do not refer in the text of your manuscript. Please ensure that you refer to Table 4 in your text; if accepted, production will need this reference to link the reader to the Table.**

- **Response:**

Thank you for noting this. This table was removed to improve the word count of the manuscript and reduce any dual publication.

8. **Please include captions for your Supporting Information files at the end of your manuscript, and update any in-text citations to match accordingly. Please see our Supporting Information guidelines for more information:** [**http://journals.plos.org/plosone/s/supporting-information.**](http://journals.plos.org/plosone/s/supporting-information)

- **Response:**

Captions for the supporting files were included at the end of the manuscript according to the PLOS guideline.

**Additional Editor Comments (if provided):**

**This is an interesting paper with great potential. The two reviewers have provided detailed feedback that can be used to improve the paper further. Please review these comments carefully as you work on a revision.**

**Reviewer #1: This is an interesting paper, well-written and comprehensive. However, it is also a very long paper!!! The authors have unnecessarily described the context and background as though it is a mini thesis which much duplication as well. I find the detailed description of the frameworks repetitive- perhaps more than sufficient in the methods but not that necessary in the background as this is not a thesis... detailed comments below.**

- **Response:**

Thank you for your review and comments. After careful revision, I have tried to remove all duplication where I could see it. I have also edited the literature/background sections and discussion to reduce the word length and address the reviewers’ comments.

**Introduction**

- **I would recommend the authors to integrate and shorten the background and introduction pieces together, the depth of information is quite overwhelming, which takes kills the enthusiasm for the findings of this study-the main point of this paper, in my view.**
- **Response:**

Thank you, this was done. The literature and background sections were edited to be one section.

**Methods**

- **The first 2 paragraphs under setting seem to belong in the introduction/background section too. Other than that, the methods are well-described!**
  - **Response:**

This was edited as per suggestion.

- **In Table 1, I'm unclear about the sample groups from number 7-12: are these neither from rural/urban, which setting do they come from? are they exclusive from the sample groups. 1-6? in other words, are the sample groups 1-6 not in a relationship? not married or single? have children???? the classification/categorization is quite confusing...e.g. the authors included young females from rural and urban, 20-34 yrs old, and also have the same age group single, married/in a relationship, and with no children (18-49 yrs)- are they different from the ones described in 1-6 and if so, how?**
  - **Response:**

I have removed the table and written the group descriptions to help clarify. To clarify the participant breakdown – in total 127 participants participated in this study in SA. Of the 127 participants, 16 were health care providers and 8 in each FGD which were divided according to professional ranking.

There were also 8 key informant IDIs.

The female community participants were first divided between the urban and rural settings. Three urban groups consisted of women aged 35 to 49 years (adults), 20 to 34 years (young adults), and 15 to 19 years (adolescents). These three groups were matched for age in the rural setting.

Three additional FGD groups were then held with a group of unmarried women (20 to 34 years), a group of married/in-union women (20 to 34 years), and a group of women who do not yet have children (18 to 49 years). These three groups were recruited from both the rural and urban areas.

All the FGD participants were unique for each group i.e., no participant was interviewed twice.

The 3 male FGD groups consisted of men aged 35 to 49 years (adults), 20 to 34 years (young adults), and 15 to 19 years (adolescents) respectively and were a mix from the rural and urban areas.

- **Only the applicable theoretical framework should be described in the methods section, concisely as well.**
  - **Response:**

Thank you, this section was edited to only refer to the applicable theoretical framework. After revising the manuscript, I noted how this can be confusing for some readers.

**Results**

**-Very comprehensive and interesting findings! In general, I like how they are presented - following on the framework structure and flow logically.**

- **The theme on implementation policy is very thin- I'd consider that part in the discussion section rather than a stand-alone theme.**
  - **Response:**

Thank you for this comment. Based on your review and the comment from reviewer 2, I decided to remove policy implementation as a theme and to rather discuss it under the discussion section.

- **Insert figure 2 has nothing to do with clinic, only a shop shown there.**
  - **Response:**

Yes, some male participants only drew shops as their source of contraception. This drawing was included to show this finding.

**Discussion**

- **Nice discussion as well, especially the policy part! However, I would suggest that is framed in a "policy implication" perspective after discussing the above findings.**
- **Perhaps no need for the "theoretical framework" structure here- just discuss the findings!**
- **It is also relatively long!**
  - **Response:**

Thank you for the suggestions made. On revision, I have edited the discussion to reduce the length and make it more concise. Please see the update discussion section.

- **Notwithstanding the framework within which this paper was conceived on, it is surprising to see the most recent "research" related reference used by the authors from 2019- surely more and relevant research has been published between 2019 and 2021 in relation to access, uptake and use of modern contraceptives. So I would challenge the authors to revisit the literature and update their references...**
  - **Response:**

Thank you for this comment. On review, there really is very limited relevant literature about access to contraception in SA – and none which are more recent in publication date. This highlights the importance of this paper as an update to what was published in the past. I also wanted to keep the word length shorter as the paper is already quite long, so I only included literature that is absolutely relevant to this paper.

**Reviewer #2: General**

**This paper is interesting and has a rich data set. Overall some of the data needs to be reviewed and expanded in the findings and discussion to adequately apply a social constructionist framework. The paper needs a minor grammar edit and a review of spelling. See abstracts in annotated paper review for a few examples.**

- **Response:**

Thank you for the review of the paper. I apologize for the grammatical errors which were updated and corrected. I have revised the results and discussion section to bring out the social constructionist stance, but I am constrained by an already lengthy manuscript to fully explore this issue. In addition, the social constructionist stance can also be appreciated from a methodological point of view, as both health care providers, key informants, and community participants’ voices were included in the findings. This allowed for each grouping, especially the divide between the health care providers and community members, to be elucidated without singling out which was more important.

Please see the track change version of the paper for the edits and corrections made.

**Introduction**

**The section on the critique of the access frameworks needs to be tighter (lines 72-81). See comments on paper.**

- **Response:**

Thank you for your comments. I have updated the theoretical framework section. Please see the manuscript for the updated text.

**Methods**

**The methods should more fully discuss the use of drawings; why they were used, how were they set up and used in the facilitation of the FGDs, and how were they analysed in relation to the coded transcripts. Were they only used to comment on access to services and geographical barriers?**

- **Response:**

Thank you for the comment. The drawings were an additional method to collect individual data within the group setting. Using this method was quite effective in allowing individual participants to share their views on accessing contraception. Overall, the drawings were quite simple and reflected what was asked: where their source of contraception was, how they got there, and how long it took.

The participants enjoyed making the drawings - it served as a bit of a break in the FGD since these FGDs were rather long.

I have added some text in the methodology section that expands a bit on the drawings. The drawings were analysed in the same manner as the rest of the data – the codebook was used, and appropriate codes were applied. These codes mainly consisted of accessibility codes, such as distance, source of contraception, time travelled, and mode of transport.

**Findings**

**The finding related to policy is weak. What more did the data say? How was integration of services experienced by women? This is discussed later but could also be explored or at least cross-referenced as it reflects the reality between policy and implementation.**

- **Response:**

Based on this comment and the comments from reviewer 1 I decided to remove policy as a theme and rather bring it up in the discussion. Policy and implementation truly affected all the areas of access and were challenging to isolate. I attempted to integrate policy discussions throughout the discussion and especially to highlight how integration impacted women.

**The use of the drawing as a basis for Table 5 and then statistics related to the drawings seems to me a rather static way of using the drawings – would you have not got the same data had these been asked in the focus groups? Were there other things that people drew that supplemented the data and helped with a deeper understanding (social construction of meaning).**

- **Response:**

A key challenge with using a visual data collection technique in qualitative research is the question of whether the same results could not be obtained by a different method.

According to the literature, the only true way to do that is to set up a rigorous experiment. For this study, the aim was not to prove the validity of one method over another but rather to obtain an updated perspective on accessing contraception. Most of the data were obtained from the interview transcripts but the drawings allowed for an individual data collection method in a group setting. This eliminated the need to repeat the data collection process. It also served as a ‘break’ during the discussions allowing the participants to interact freely with the interviewers and each other.

Could the same data have been obtained during the FGD itself by merely asking the question about how they experienced accessing contraception? Perhaps, but the individual experience would have been lost. Each participant drew their own experience, and their own specific challenges when going to collect their contraception. That level of detail would have been lost in a group discussion since not every participant responds to every question asked. It was important for this study to gain an understanding of how, where, and what challenges participants experienced when they wanted to access contraception. This was especially the case because not all the participants were current or ever contraceptive users and we wanted to understand where the barriers may lie.

**See other comments on marked-up paper for specific areas that need strengthening.**

- **Responses to comments in PDF:**
- All grammar and spelling errors were corrected.
- **Lines 72 to 81 –** moved to the theoretical framework section to improve the flow of the paper. The critique was emphasized in the updated section.
- **Lines 137 – 138 –** Sentence was updated as suggested.
- **Lines 186 – 188 -** Thank you for the comment. The drawings were an additional method to collect individual data within the group setting. Using this method was quite effective in allowing individual participants to share their views on accessing contraception. Overall, the drawings were quite simple and reflected what was asked: where their source of contraception was, how they got there, and how long it took.

The participants enjoyed making the drawings - it served as a bit of a break in the FGD since these FGDs were rather long.

I have added some text in the methodology section that expands a bit on the drawings.

- **Line 296** – Thank you for the comment. As per the reviewers’ suggestion, I have decided to remove the policy theme from the thematic results and to rather discuss it in the discussion since the influence of policy and policy implementation was so pervasive in the data set.
- **Line 433 – 434** – Thank you for the comment. The sentence was edited to include the reviewer’s suggestion.
- **Line 452 – 456** – In the results, the participants explain that the focus on the family planning services is lost due to the clinics being overburdened with a variety of clients. Both the community members and the health care providers noted this. This topic was elaborated on a bit in the updated discussion.
- **“Your data also shows that integration of services at PHC level (policy directed) has possibly (unintentionally) resulted in lower levels of access- as you describe waiting times, missed visits etc etc” –** Thank you for the comment. This was highlighted in the discussion.
- **Lines 600 –** This sentence was in reference to the literature that was cited. The sentence was removed for clarity.
- **Lines 616 – 621 -** Thanks for this comment. I agree that the issues of agency, autonomy and economic power play an important role in contraceptive use (or non-use). The discussion was updated to reflect these important points.
- **Lines 635 – 640 -** Thank you for the comment. We agree this reflects the limits of policy implementation and have added a sentence to help clarify.
