# Supplementary material for: Access to public sector family planning services and modern contraceptive methods in South Africa: A qualitative evaluation from community and health care provider perspectives
Source: PLoS One. 2023 Mar 17;18(3):e0282996. doi: 10.1371/journal.pone.0282996 (PMC10022780; doi:10.1371/journal.pone.0282996)
Supplement: Attachment — Submitted filename: Responses to reviewers 09Jan23.docx [file pone.0282996.s010.docx]

| **Reviewer comment** | **Response** |
| --- | --- |
| Reviewer 3 |  |
| This paper attempts to fill a gap which is the lack of use of modern contraceptive methods in KwaZulunatal, South Africa. The paper is well written and qualitative data shed some light on “access” to family planning methods from a broader perspective that include five elements according to a theoretical framework. However, these five elements needs to be well defined from the start. Unfortunately, data don’t bring much new insights in particular on the acceptability issue including the issue of secondary effects which is reported worldwide as the main reason for lack of uptake or continuation. The social factors or what is called the “social constructionism” lens used to view the data could be more prominent and data presented and discussed in light of social determinants (unmarried or single women or other vulnerable groups). | Thank you for your review.  This study specifically focussed on access to contraception in the South African public sector clinics. No previous studies have been conducted that specially focussed on access to contraception using the five A’s framework. The aim of the study, and this manuscript, was to uncover the barriers and enablers to accessing contraception in this setting. The aim was not to further the definition of access or its components.  In the previous draft of this manuscript, reviewer 2 suggested that the definitions are not necessary and lengthen the overall paper. The theoretical framework section was therefore shortened and the definitions removed. I have, however, reinserted these definitions under the theoretical frameworks section again. Please see lines 132-139  The acceptability component was explored in detail in another publication as the data fit more accordingly under the quality of care framework. Please see this publication for further reading:  Kriel, Y., Milford, C., Cordero, J. P., Suleman, F., Steyn, P. S., & Smit, J. A. (2021). Quality of care in public sector family planning services in KwaZulu-Natal, South Africa: a qualitative evaluation from community and health care provider perspectives. *BMC Health Services Research*, *21*(1). <https://doi.org/10.1186/s12913-021-07247-w>  The social constructionist stance was used to analyse and review the data. Due to word length limitations and revisions requested from the previous reviewer, we are unable to fully explore the social constructionist viewpoint in this paper. However, important social structures are highlighted such as the various group categories (married, unmarried, no children, and men.). The social constructionist stance was very helpful as a lens through which to view the data, especially since there are significant social hierarchies between the groups of participants in the study. For instance, we were consciously aware of the power dynamics that can exist between healthcare providers and community members and that the healthcare provider’s view can sometimes override that of community members/clients. This power dynamic also exists between the adult and adolescent participants.  Therefore, social constructionism provided guidance in minimising biases in our reporting. |
| 1. There is some confusion or contradictions along the text with the term “access” despite the fact that you present a framework you will use in this study and you define access accordingly. It should clearly presented from the start in the theoretical framework section after defining access, what each term (Five interrelated components of access used in this framework, namely accessibility, availability, affordability, accommodation, and acceptability) will entail, stick to that and be consistent. | Thank you, the definitions were re-inserted.  Careful attention was paid throughout this study to the definitions of the components of access. |
| Page 2 lines 47-48 it is mentioned :” This study illustrated the importance of examining access as a holistic concept and not just the availability of services or methods.” I don’t think that access is commonly related or limited to the availability of contraceptive methods; on the contrary access refers primarily to geographical and financial access. I would remove or reformulate that sentence. Moreover in the introduction page 4 lines 96-100: you had a contradictory statement as a justification of the paper: “Only focusing on affordability and physical access contributes to an inaccurate understanding of access. Other sources have identified additional barriers, including poor quality of care (QoC), limited variety of modern contraceptive methods, fragmented integration of sexual and reproductive health (SRH) services with PHC services, ailing infrastructure, shortage of trained nurses, poor attitudes of nurses, and stigma (5, 15-19)” | In our read of the literature, access was commonly equated with availability. The sentence was updated.  This sentence is in reference to the categories used in the DHS survey to measure access. |
| Results : Line 280 Accessibility: “Three sub-themes emerged from this theme – geographic access to current contraceptive sources, infrastructure, and preferred source of contraceptive methods.” Then later line 296 “ Infrastructure : HCP participants described the availability and accessibility of contraceptive methods at their healthcare facility related to the available infrastructure. | This sentence was updated. Please see lines 306-308. |
| Again in the Conclusion there is some contradiction and confusion regarding the meaning of access: It is stated line 616 “Overall, the participants were satisfied with the accessibility and affordability components” and later line 623 “ Men described the financial burden of accessing contraception and highlighted the importance of having an alternative source of contraception in the communities that are easily accessible.” Please clarify and reformulate Please define the terms from the start and harmonize the text and the abstract. | The first sentence refers to the overall study findings. However, male participants added a specific view on the cost of accessing contraception. This cost is not often recognised or reported. It is, therefore, important to point out these differences.  The sentence was slightly altered for clarity. |
| 2. Page 5 : the second paragraph (Lines 105-120) is long and could be quite shortened if you start the paragraph by the following : “Despite the enabling policies and restructuring, the SA public health sector has struggled to 118 make real gains in improving access to services, including FP service (20, 29, 30). | Thank you for the suggestion, however, the preceding sentences provide the context with which to justify the last statement. |
| 3. Introduction: Although your study focuses on the public sector, it would be indicated to mention in the introduction (and also in the settings- eThekwini District of KwaZulu-Natal SADHS results related to what are the main sources of family planning in SA, in particular: % of public vs. private services? , and most preferred sources of contraception among young people and adults ? and most contraceptive methods used in SA and KwaZulu-Natal. | A paragraph was inserted regarding the sources of contraception, see lines 105 - 119 |
| 4. Page 8 “CPR, “for KZN has consistently fallen below the national median, with the 2018/19 CYPR 154 being 44.4%” then later “The CYPR for eThekwini is also below the national average 159 at 44.4% for 2018/2019” please clarify for consistency.Page 8 lines 155: Study settings: Please add HIV prevalence (then the total population affected by HIV) in KwaZulu natal. | Thank you for seeing this error – the second sentence is an error and was deleted.  Please see lines 175-179 for the HIV statistics. |
| 5. Page 8 line 171 “Twelve of these FGDs were with community participants (n= XX) , and two with HCP (n= XX) ” I think you removed the table (duplication of data presentation) but please mention the total number of community members participants in FGD/ total HCP in FGD; and total IDI “ IDIs were conducted with eight key informants (KIs) (n= XX) . | Thank you for noticing this omission. The reviewer 2 suggested a removal of the tables that describe the participant characteristics to reduce data duplication. I have re-inserted the total numbers for each category. |
| 6. Line 126: “Theoretical framework” can fit in the Methods section and should not be a standalone section. Even if you present other frameworks you should stick to one (and I think this is what you did) and the sub-title should be called “theoretical framework” (not frameworks) . | In the previous review, reviewer 2 requested that the theoretical framework section be separate. I have made the edits as you suggested and moved it back to the methodology section. |
| 7. Under the theme Affordability you include ability (Line 368) Ability to access FP service and contraceptive methods. The first sub-theme is the user’s ability to access SRH services of their own choice – referred to as ‘individual power’ by community participants. The participants described their limited financial situation (I would add - as one factor) constraining their ability to obtain contraception. In my view ability or individual power is a much broader theme and the financial constraint is only one factor. Did other ability factors emerge from the interviews? | Thank you for your comment.  I agree that ability could be regarded as a broader theme. However, specific to this data, the participants referenced their financial situation and ability to pay, as key factors that influence their access to services.  I have edited the theme heading to clarify that in this paper I am referring to the financial/economic ability to access services. |
| 8. The acceptability component is not addressed: why? And if no material on that it should be mentioned in the study limitations. | Thank you for this comment.  In the overall project, the Quality of Care was also examined as a separate topic. Considering the definition of the acceptability component it was more fitting to report the acceptability data in a separate paper under the quality of care framework. This is highlighted in lines 300-301. For further reading on this, please see out other publication: (Kriel et al., 2021)  Kriel, Y., Milford, C., Cordero, J. P., Suleman, F., Steyn, P. S., & Smit, J. A. (2021). Quality of care in public sector family planning services in KwaZulu-Natal, South Africa: a qualitative evaluation from community and health care provider perspectives. *BMC Health Services Research*, *21*(1). <https://doi.org/10.1186/s12913-021-07247-w> |
| 1. Abstract - methods: please provide the total number of participants - community members (n=XX) and health care providers (n=XX) | Thank you, the total numbers were added. |
| 2. Page 4 lines 56-58: “…Sexual and Reproductive Health (SRH)” and “Human Rights” : please avoid to use too many capital letters for names (even before using an acronym) unless they are organizations or recognized as needing a capital letter. | Thank you, the text was updated. |
| 3. Discussion: you could possibly discuss the complexity of the term access, its definitions or different meanings? | The aim of this paper was not to address the complexity of access as a concept, however, I added a short paragraph in the discussion which highlights this. Please see lines 509-524. |
|  |  |
| **Reviewer 4 -** Reviewer #4: This is a revised version. This research has been well done. The reviewers from the previous round made important comments, but the authors seem to me to have replied adequately. I have only a few small comments or suggestions: |  |
| 1. The section titled ‘analysis’ is about data-collection and data-analysis. I suggest to split this into two sections. | Thank you for the suggestion. The sections were split and the headings were changed. |
| 2. Is it possible to reflect on the fact that selection bias can occur during the recruitment of the participants? Can this influence the results? | Thank you for this comment. The selection bias was minimised in this study as the enrolment consisted of community members who may or may not have been contraceptive users. Random community members were recruited into this study instead of only clinic attendees. However, a small bias may well be present as only members from two communities were selected. A short paragraph was added to highlight this – please see lines 638-641. |
|  |  |
