# Supplementary material for: Access to public sector family planning services and modern contraceptive methods in South Africa: A qualitative evaluation from community and health care provider perspectives
Source: PLoS One. 2023 Mar 17;18(3):e0282996. doi: 10.1371/journal.pone.0282996 (PMC10022780; doi:10.1371/journal.pone.0282996)
Supplement: Attachment — Submitted filename: Response to reviewers 27Feb23.docx [file pone.0282996.s011.docx]

Responses to reviewers: 27 Feb 2023

1. Lines 120-121: “On a national policy level, the SA government is dedicated to improving access to SRH services and modern contraception by committing to the goals set out by FP2020 (24).” Have these goals been carried over by FP2030? Please edit this sentence reflecting the transition from FP2020 to FP2030.

Response: - Sentence was edited to update to FP2030

2. Lines 129-130: “However, barriers to accessing health care services (as mentioned above) must be addressed for integration to be effective (WHO, 2008).” Please use the Vancouver style to reference this WHO document as has been done in the rest of the manuscript.

Response: Thank you for noticing this. The reference was updated to Vancouver style.

3. Lines 175 & 706: Please edit “The couple’s year of protection rate...” to read “The couple-years of protection rate...”.

Response: Thank you for noticing this error. Update made.

4. Lines 192-193: For clarity, I suggest the number of FGDs (n=14) be written before the number of individuals that participated in the FGDs (n=127). Please consider this suggestion in the abstract as well.

Response: Thank you, this edit was made. It does add some clarity.

5. Lines 229-230: Please delete the word “study” so the sentence reads, “During the FGDs and IDIs, participants were asked to explore their views about the uptake of modern contraception.”

Response: Thank you, the word study was deleted. This does make the sentence more clear.

6. Lines 279-280:  I would argue that 53% is not “most”. I suggest the authors consider “over half” or “Fifty-three percent” or another phrase or word.

Response: Thank you for the suggestion, the edit makes sense.

7. Please take a look at the references listed below and edit accordingly. I feel each of them needs a minor edit, such as a space, the insertion of the journal volume or number, or the deletion of a duplicated year. References: 18 (lines 761-762), 24 (lines 775-776), 37 (lines 808-809), 40 (lines 815-816), 43 (lines 822-823), 46 (line 831), 56 (line 855), 62 (line 872), 65 (line 879).

Response: Thank you for noticing these errors. I have updated the references and tried to correct and edit for spacing. During the format editing by the journal the spacing should also resolve.

Reviewer #4: I still have 2 comments:
1. Lines 229 – 251 are about data collection and not data-analysis.

Response: I have updated the heading to reflect data collection and analysis.

2. It is unclear how the participants were recruited. On lines 193 – 194 the authors wrote: “Purposive snowball sampling was used as the recruitment strategy”, while on lines 638 – 639, they wrote: “While the selection of the community and healthcare participants into this study was random a selection”.

Response: Thank you for pointing that out. Participants were selected using a snowball technique, which was purposive due to the categories required for the study. The sentence in concluding paragraph was inaccurate. I have updated that sentence to clarify the potential for study bias that could have been introduced into the data due to the selection of participants. I hope this clarifies the issue.
